# Metabolic adaptations rewire CD4^+^ T cells in a subset-specific manner in human critical illness with and without sepsis

**DOI:** 10.1038/s41590-025-02390-6

**Published:** 2026-01-15

**Authors:** Matthew T. Stier, Allison E. Sewell, Erin L. Mwizerwa, Chooi Ying Sim, Samantha M. Tanner, Casey M. Nichols, Heather H. Durai, Erin Q. Jennings, Paul Lindau, Erin M. Wilfong, Sarah N. Obeidalla, V. Eric Kerchberger, Dawn C. Newcomb, Julie A. Bastarache, Lorraine B. Ware, Jeffrey C. Rathmell

**Affiliations:** 1 Division of Allergy, Pulmonary and Critical Care Medicine, Department of Medicine, Vanderbilt University Medical Center, Nashville, TN USA; 2 Vanderbilt Center for Immunobiology, Vanderbilt University Medical Center, Nashville, TN USA; 3 Department of Pathology, Microbiology and Immunology, Vanderbilt University Medical Center, Nashville, TN USA; 4 Division of Hematology and Oncology, Department of Medicine, Vanderbilt University Medical Center, Nashville, TN USA; 5 Department of Medicine, Vanderbilt University Medical Center, Nashville, TN USA; 6 Division of Rheumatology and Immunology, Department of Medicine, Vanderbilt University Medical Center, Nashville, TN USA; 7 Department of Biomedical Informatics, Vanderbilt University Medical Center, Nashville, TN USA; 8 Department of Cell and Developmental Biology, Vanderbilt University, Nashville, TN USA; 9 Ben May Department for Cancer Research, Ludwig Center at the University of Chicago, Chicago, IL USA

**Keywords:** Inflammatory diseases, Adaptive immunity, Lymphocytes

## Abstract

Metabolic and immunologic dysfunction, including pathological CD4^+^ T cell immunosuppression, are archetypal in critical illness, but whether these factors are mechanistically linked remains incompletely defined. Here we characterized the metabolic properties of human CD4^+^ T cells from critically ill patients with and without sepsis and healthy adults. CD4^+^ T cells in critical illness showed subset-specific metabolic plasticity, with regulatory T (T_reg_) cells preferentially acquiring glycolytic capacity that associated with sustained cellular fitness and worsened clinical illness. Adapted T_reg_ cells were more metabolically flexible and stabilized suppressive markers FOXP3 and TIGIT under mitochondrial stress. Single-cell transcriptomics suggested reactive oxygen species (ROS) and kynurenine metabolism as drivers of T_reg_ cell remodeling. Subsequent inhibition of ROS and kynurenine metabolism attenuated glycolytic adaptation and suppressive rewiring, respectively, in T_reg_ cells. These findings indicate that metabolic dysfunction was a contributor to CD4^+^ T cell remodeling in critical illness and suggest avenues to restore effective immunity.

## Abstract (web summary)

Rathmell and colleagues show that metabolic reprograming of regulatory T cells is associated with severity of critical illness in patients with and without sepsis.

## Main

Critical illness (CI) requiring intensive care unit (ICU) admission and life support due to severe organ failure affects over 5 million patients annually in the USA^1^. Among the most common ICU diagnoses is sepsis, a dysregulated host response to infection causing end-organ injury^2^. Sepsis accounts domestically for over 200,000 deaths per year and more than US$24 billion in healthcare expenditures^3,4^. Current management includes antimicrobials and/or surgical source control to address the inciting infection alongside supportive care measures, including vasopressors, mechanical ventilation and renal replacement therapy, to sustain vital functions while host repair mechanisms mediate recovery. Despite decades of research, no targeted therapies exist to effectively treat the pathophysiology of sepsis^5^.

Immunologic dysfunction is a hallmark of sepsis. Early sepsis features excessive inflammation with increased levels of proinflammatory cytokines including tumor necrosis factor (TNF) and interleukin-1β (IL-1β) (referred to as a ‘cytokine storm’) that contribute to tissue injury^6^. In parallel, a compensatory but disproportionate immunosuppressive response develops and can continue beyond the acute phase. This impaired immune state predisposes patients to secondary infections, persistent organ dysfunction, hospital readmissions and death^7–9^. CD4^+^ T cells are central to this immunosuppressive remodeling, with effector CD4^+^ T cells acquiring an ‘exhausted-like’ phenotype characterized by high PD-1 and LAG-3 expression, attenuated TNF and interferon-γ (IFNγ) secretion and increased apoptosis, whereas suppressive T_reg_ cells are relatively preserved^10–12^. The severity of this cell-mediated immune impairment is underscored by the frequent detection of cytomegalovirus (CMV), Epstein–Barr virus (EBV) and herpes simplex virus (HSV) in critically ill patients^13^ at rates similar to solid organ transplant recipients receiving T cell immunosuppressants^14^. Although these phenotypes are consistently observed, the pathways underlying CD4^+^ T cell immunosuppression in human CI and sepsis remain incompletely understood.

Metabolic dysfunction is pervasive in CI, including sepsis^15^. The metabolic state of immune cells is a mechanistic determinant of their fate and function. Immunometabolism is central to the pathogenesis of obesity, autoimmunity, cancer and SARS-CoV-2 infection^16,17^ and metabolic reprogramming has emerged as a promising therapeutic target in these conditions^18^. Previous human studies from individuals with sepsis have reported abnormalities in mitochondrial complex I and V activities and broad impairments in glycolysis and oxidative phosphorylation among bulk T lymphocytes^19,20^; however, the overall role of CD4^+^ T cell immunometabolism in sepsis and other CI syndromes remains understudied, particularly stratified by CD4^+^ T cell subset.

In this study, we used biospecimens from critically ill patients to comprehensively profile the metabolic dependencies and capacities of CD4^+^ T cell subsets. We showed that reactive oxygen species (ROS)-driven glycolytic reprogramming, together with increased kynurenine metabolism, heightened metabolic plasticity in T_reg_ cells and promoted their resilience and suppressive function at the expense of conventional CD4^+^ T cell subsets. This enhanced glycolytic capacity in T_reg_ cells was additionally associated with worse clinical outcomes.

## Results

### ICU-derived T_reg_ cells have higher glycolytic capacity

**Fig. 1:**
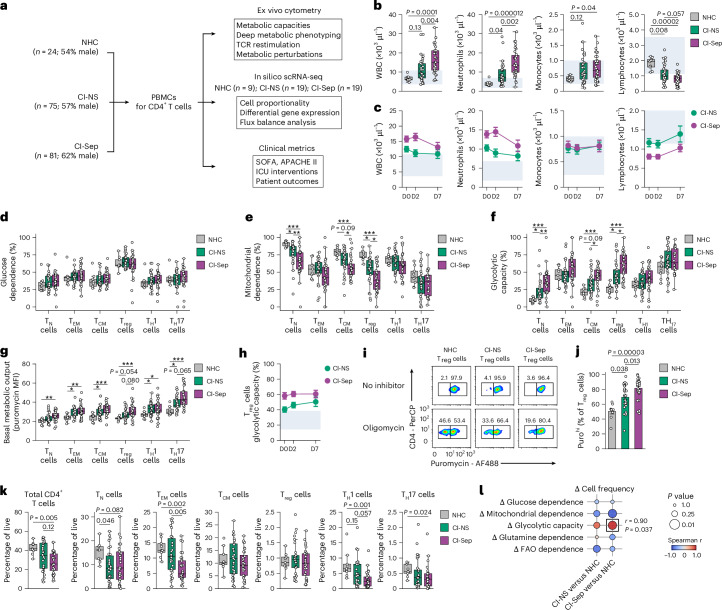
CD4^+^ T cells exhibit subset-specific metabolic adaptations in the context of critical illness with and without sepsis. **a**, Schematic depicting the study cohort including NHCs (*n* = 24, 54% male), CI-NS (*n* = 75, 57% male) and CI-Sep (*n* = 81, 62% male) and the analytical pipeline. **b**, Peripheral blood cell counts of total white blood cells (WBCs), neutrophils, monocytes and lymphocytes in NHCs (*n* = 9), CI-NS (*n* = 34) and CI-Sep (*n* = 35) at day 2 post-ICU admission. **c**, Peripheral blood cell counts of total WBCs, neutrophils, monocytes and lymphocytes from at day (D) 0, 2 and 7 post-ICU admission in CI-NS (day 0, *n* = 43; day 2, *n* = 34; day 7, *n* = 12) and CI-Sep (day 0, *n* = 51; day 2, *n* = 35; day 7, *n* = 20). Shaded region represents the assay normal reference range. **d**–**f**, Glucose dependence (**d**), mitochondrial dependence (**e**) and glycolytic capacity (**f**) in CD45RA^+^CCR7^+^CD4^+^ T_N_ cells, CD45RA^−^CCR7^−^CD4^+^ T_EM_ cells, CD45RA^−^CCR7^+^CD4^+^T_CM_ cells, CD25^+^FOXP3^+^ T_reg_ cells, CXCR3^+^CCR6^−^ T_H_1 cells and CCR4^+^CCR6^+^ T_H_17 cells determined by puromycin-incorporation assay at day 2 post-ICU admission in NHCs (*n* = 9), CI-NS (T_N_, T_EM_, T_CM_ and T_reg_ cells, *n* = 30; T_H_1 and T_H_17 cells, *n* = 29) and CI-Sep (T_N_, T_EM_ and T_CM_ cells, *n* = 32; T_reg_ cells, *n* = 30; T_H_1 cells, *n* = 31; T_H_17 cells, *n* = 24). Displayed as percentage of the maximum puromycin incorporation without metabolic inhibitors. **g**, Basal metabolic output determined by the geometric MFI of puromycin in the absence of metabolic inhibitors in CD4^+^ T_N_, T_EM_, T_CM_, T_reg_, T_H_1 and T_H_17 cells isolated from NHCs (*n* = 9), CI-NS (T_N_, T_EM_, T_CM_ and T_reg_ cells, *n* = 30; T_H_1 and T_H_17 cells, *n* = 29) and CI-Sep (T_N_, T_EM_ and T_CM_ cells, *n* = 32; T_reg_ cells, *n* = 30; T_H_1 cells, *n* = 31; T_H_17 cells, *n* = 24) at day 2 post-ICU admission. **h**, Time course of glycolytic capacity in T_reg_ cells at day 0, 2 and 7 post-ICU admission from CI-NS (day 0, *n* = 36; day 2, *n* = 30; day 7, *n* = 12) and CI-Sep (day 0, *n* = 32; day 2, *n* = 30; day 7, *n* = 9). Shaded region represents an estimated normal range using NHC T_reg_ cells as in **f** and plotting the mean ± 2 × s.d. **i**,**j**, Representative flow cytometry plots (**i**) and quantification of puromycin incorporation (**j**) in T_reg_ cells isolated at day 2 post-ICU admission from NHCs (*n* = 9), CI-NS (*n* = 30) or CI-Sep (*n* = 30) and treated with or without oligomycin for 70 min, with puromycin present for the last 40 min. Displayed gates denote Puro^hi^ (right) and Puro^lo^ (left) populations. Percentages are of the T_reg_ cell parent gate. **k**, Frequency of total CD4^+^ T cells and T_N_, T_EM_, T_CM_, T_reg_, T_H_1 and T_H_17 cells of all live cells isolated from NHCs (*n* = 9), CI-NS (*n* = 30) or CI-Sep (*n* = 32) at day 2 post-ICU admission. **l**, Two-sided spearman rank correlation between the percentage change in the population mean frequency of live cells for CD4^+^ T_N_, T_CM_, T_reg_, T_H_1 and T_H_17 cells in CI-Sep or CI-NS compared with NHCs and the population mean change in glycolytic capacity for CD4^+^ T_N_, T_CM_, T_reg_, T_H_1 and T_H_17 cells from CI-Sep or CI-NS compared with NHCs. The T_EM_ cell subset was excluded to prevent pseudoreplication arising from their overlap with the nested T_H_1 and T_H_17 cell subsets. Statistical analysis for **b**,**d**–**g**,**j**,**k** was performed using the Kruskal–Wallis test with Dunn’s post hoc testing. Where possible, actual *P* values are shown. In certain instances, owing to the density of the data labels, *P* values are reflected as *P < 0.05, **P < 0.01 and ***P < 0.001. Data for **c**,**h**,**j** are presented as mean ± s.e.m. Boxplots show the median with boxes bounded by interquartile range (IQR; 25–75th percentile) and whiskers extending to the minimum and maximum values within 1.5 × IQR. Each data point represents an individual donor. Source data

**Extended Data Fig. 1:**
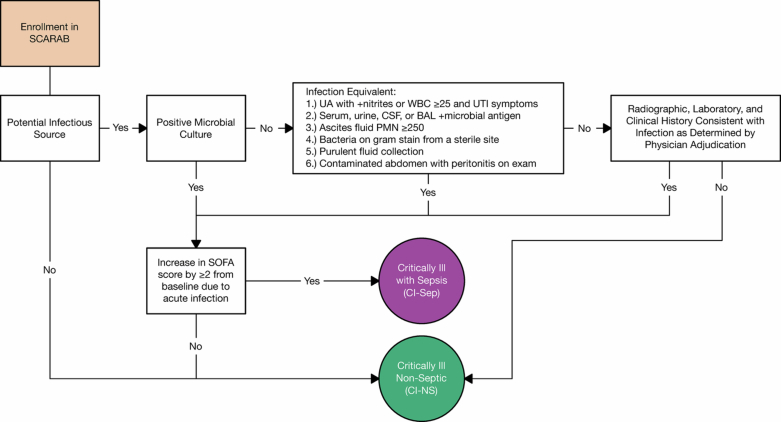
Sepsis adjudication algorithm. Adjudication algorithm used to assign sepsis status to enrolled subjects. UA = urinalysis; WBC = white blood cells; UTI = urinary tract infection; CSF = cerebrospinal fluid; BAL = bronchoalveolar lavage; PMN = polymorphonuclear cells.

We prospectively collected peripheral blood mononuclear cells (PBMCs) from 156 patients in the medical and surgical ICUs at Vanderbilt University Medical Center upon ICU admission, as well as serially, at day 2 and day 7 post-admission if available (Fig. 1a and Extended Data Table 1). Participants were adjudicated into critically ill patients who were nonseptic (CI-NS; *n* = 75, 57% male, median age 63 years, IQR 55–70) and critically ill patients with sepsis (CI-Sep; *n* = 81, 62% male, median age 65 years, IQR 56–75; Extended Data Fig. 1 and Extended Data Table 1). Nonacutely ill healthy control participants (NHCs; *n* = 24, 54% male, median age 54 years, IQR 42–58) were recruited from the community as controls. Complete blood counts from CI-NS and CI-Sep showed a mixed neutrophilic (NHC median 3.72 × 10^3^ μl^−1^, CI-NS 7.12 × 10^3^ μl^−1^ and CI-Sep 13.51 × 10^3^ μl^−1^) and monocytic (NHC median 0.42 × 10^3^ μl^−1^, CI-NS 0.57 × 10^3^ μl^−1^ and CI-Sep 0.67 × 10^3^ μl^−1^) response with marked lymphopenia (NHC median 1.92 × 10^3^ μl^−1^, CI-NS 0.99 × 10^3^ μl^−1^ and CI-Sep 0.83 × 10^3^ μl^−1^) (Fig. 1b,c), as reported^21^, a characteristic hematologic pattern in critically ill patients reflecting heightened innate immune activation and loss of circulating lymphocytes.

**Extended Data Fig. 2:**
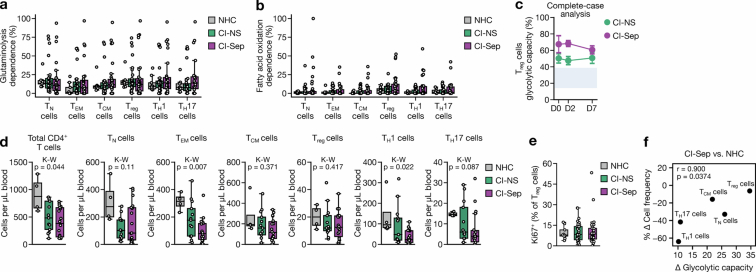
Additional features of CD4^+^ T Cells in CI. **a**,**b**, Glutaminolysis dependence (**a**) and fatty acid oxidation (FAO) dependence (**b**) in CD4^+^ T_N_, T_EM_, T_CM_, T_reg_, T_H_1, and T_H_17 cells determined by puromycin-incorporation assay at day 2 post-ICU admission in NHC (*n* = 9), CI-NS (T_N_, T_EM_, T_CM_, and T_reg_ cells, *n* = 30; T_H_1 and T_H_17 cells, *n* = 29) and CI-Sep (glutaminolysis dependence in T_N_, T_EM_, and T_CM_ cells, *n* = 31; FAO dependence in T_N_, T_EM_, and T_CM_ cells, *n* = 32; glutaminolysis dependence T_reg_ cells, *n* = 29; FAO dependence T_reg_ cells, *n* = 30; glutaminolysis dependence T_H_1 cells, *n* = 30; FAO dependence T_H_1 cells, *n* = 31; glutaminolysis dependence T_H_17 cells, *n* = 23; FAO dependence T_H_17 cells, *n* = 24). Displayed as percentage of the maximum puromycin incorporation without metabolic inhibitors. **c**, Complete-case analysis of glycolytic capacity in T_reg_ cells from patients with PBMC at all timepoints (day 0, 2 and 7) post-ICU admission for CI-NS (*n* = 11) and CI-Sep (*n* = 9). Estimated normal range is shaded. **d**, Cell counts of total CD4^+^ T, T_N_, T_EM_, T_CM_, T_reg_, T_H_1, and T_H_17 cells per μL blood in NHC (n = 4), CI-NS (n = 13) and CI-Sep (n = 18) at day 2 post-ICU admission. **e**, Flow cytometric quantification of the frequency of Ki67^+^ cells among T_reg_ cells in NHC (*n* = 9), CI-NS (*n* = 30) or CI-Sep (*n* = 30) T_reg_ cells at day 2 post-ICU admission. **f**, Scatter plot showing two-sided Spearman rank correlation of the percentage change in the frequency and population mean change in glycolytic capacity of CD4^+^ T_N_, T_CM_, T_reg_, T_H_1, and T_H_17 cells in CI-Sep compared to NHC, excluding the T_EM_ cell subset to prevent pseudoreplication from overlap with nested T_H_1 and T_H_17 cell subsets. Statistical analysis for a,b,d,e was performed using the Kruskal–Wallis test with Dunn’s post-hoc testing. Data for c are presented as mean ± SEM. Boxplots show the median with boxes bounded by interquartile range (IQR, 25–75th percentile) and whiskers extending to the minimum and maximum values within 1.5 x IQR. Each data point represents an individual donor. K–W = Kruskal–Wallis. Source data

To decipher the immunometabolic landscape of CD4^+^ T cells in CI-NS and CI-Sep, we used a puromycin-incorporation assay in 9 NHC, 30 CI-NS and 32 CI-Sep samples collected 48–72 h (day 2) post-ICU admission. CD4^+^ T cell subsets, including CD45RA^+^CCR7^+^ naive T (T_N_) cells, CD45RA^−^CCR7^−^ effector memory T (T_EM_) cells, CD45RA^−^CCR7^+^ central memory T (T_CM_) cells, CD25^+^FOXP3^+^ T_reg_ cells, CXCR3^+^CCR6^−^ type 1 helper T (T_H_1) cells and CCR4^+^CCR6^+^ T_H_17 cells, had similar dependence on glucose, fatty acid oxidation and glutaminolysis compared to NHCs (Fig. 1d and Extended Data Fig. 2a,b), whereas mitochondrial dependence and glycolytic capacity broadly differed between CI and NHCs (Fig. 1e,f). Compared to NHCs, CI T_reg_ cells showed the greatest increase in glycolytic capacity (median CI-NS +15%, CI-Sep +34%), whereas T_EM_ (CI-NS +2%, CI-Sep +12%), T_H_1 (CI-NS +4%, CI-Sep +9%) and T_H_17 (CI-NS +8%, CI-Sep +6%) had the least increase (Fig. 1f). These differences were higher in CI-Sep compared with CI-NS (Fig. 1f). There was broadly increased metabolic output across all CD4^+^ T cell subsets in CI compared with NHC (Fig. 1g). T_reg_ cell glycolytic capacity remained persistently raised up to day 7 of hospitalization (Fig. 1h and Extended Data Fig. 2c). Two metabolically distinct programs marked by sensitivity (Puro^lo^) or resistance (Puro^hi^) to the mitochondrial ATPase inhibitor oligomycin were detected in T_reg_ cells from NHCs, whereas CI-Sep T_reg_ cells were considerably enriched in Puro^hi^ cells (Fig. 1i,j). Collectively, although their baseline dependence on glucose was unchanged, CI T_reg_ cells showed reduced mitochondrial dependence and higher overall glycolytic capacity, indicating metabolic flexibility as part of a putative stress-adaptive program.

While lymphopenia was evident in both CI-NS and CI-Sep, the degree of attrition varied across CD4^+^ T cell subsets (Fig. 1k). At day 2 post-ICU admission, CI-Sep patients showed reduced frequencies of conventional CD4^+^ T (T_conv_) cell) subsets relative to NHCs, including T_N_ cells (median 9% versus 16%), T_EM_ cells (5% versus 13%), T_H_1 cells (2% versus 6%) and T_H_17 cells (0.3% versus 0.6%). By contrast, the frequency of T_reg_ cells was preserved in CI-Sep (0.8%) compared with NHC (0.9%) (Fig. 1k). Estimated absolute cell counts showed a similar tendency in a subset of 4 NHCs, 13 CI-NS and 18 CI-Sep (Extended Data Fig. 2d), aligning with reports that stable or increased T_reg_ cell frequencies in sepsis reflect a loss of T_conv_ cell subsets^10^. T_reg_ cell proliferation was similar in CI compared to NHCs (Extended Data Fig. 2e). Increased acquisition of glycolytic capacity, represented as the change in CI-Sep versus NHC mean glycolytic capacity for each CD4^+^ T cell subset, directly associated with CD4^+^ T cell subset resistance to attrition (Fig. 1l and Extended Data Fig. 2f), consistent with metabolic plasticity as a determinant of cellular persistence in CI and sepsis that ultimately favors T_reg_ cells over CD4^+^ T_conv_ cells.

**Fig. 2:**
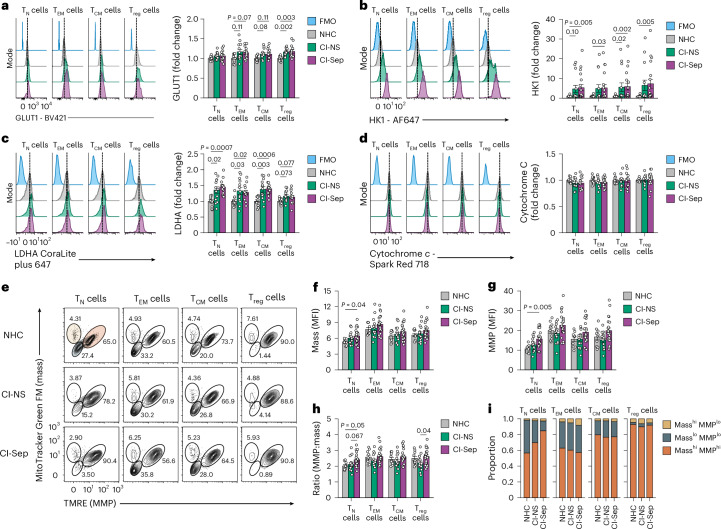
Glycolytic and mitochondrial characteristics of CD4^+^ T cell subsets in CI with and without sepsis. **a**–**d**, Representative flow cytometry histograms (left) and quantification (right) of expression of GLUT1 (**a**), HK1 (**b**), LDHA (**c**) and cytochrome *c* (**d**) in CD4^+^ T_N_, T_EM_, T_CM_ and T_reg_ cells from day 2 post-ICU admission. Shown as fold change of the MFI in CI-NS (GLUT1, LDHA and cytochrome *c*, *n* = 13; HK1 *n* = 14) and CI-Sep (GLUT1, LDHA and cytochrome *c*, *n* = 14; HK1 *n* = 19) relative to NHCs (*n* = 11). **e**, Representative flow cytometry plots of mitochondrial mass measured by MitoTracker Green FM and MMP measured by tetramethylrhodamine ethyl ester (TMRE) in NHCs, CI-NS and CI-Sep CD4^+^ T_N_, T_EM_, T_CM_ and T_reg_ cells at day 2 post-ICU admission. Gated on Mass^hi^MMP^hi^ (orange-shaded gate), Mass^lo^MMP^lo^ (steel blue-shaded gate) and Mass^hi^MMP^lo^ (yellow-shaded gate). **f**,**g**, Quantification of mitochondrial mass (**f**) and MMP (**g**) as in **e** among Mass^hi^MMP^hi^CD4^+^ T_N_, T_EM_, T_CM_ and T_reg_ cells in NHCs (*n* = 10), CI-NS (*n* = 16) and CI-Sep (*n* = 22). **h**, Ratio of MMP:mitochondrial mass as in **f**,**g** in CD4^+^ T_N_, T_EM_, T_CM_ and T_reg_ cells from NHCs (*n* = 10), CI-NS (*n* = 16) and CI-Sep (*n* = 22). **i**, Proportion of Mass^hi^MMP^hi^, Mass^lo^MMP^lo^ and Mass^hi^MMP^lo^ cells in CD4^+^ T_N_, T_EM_, T_CM_ and T_reg_ cells from NHCs (*n* = 10), CI-NS (*n* = 16) and CI-Sep (*n* = 22). Statistical analysis for **a**–**d**,**f**–**h** was performed using the Kruskal–Wallis test followed by Dunn’s post hoc testing. Data for **a**–**d**,**f**–**h** are presented as mean ± s.e.m. Each data point represents an individual donor. FMO, fluorescence minus one control. Source data

**Extended Data Fig. 3:**
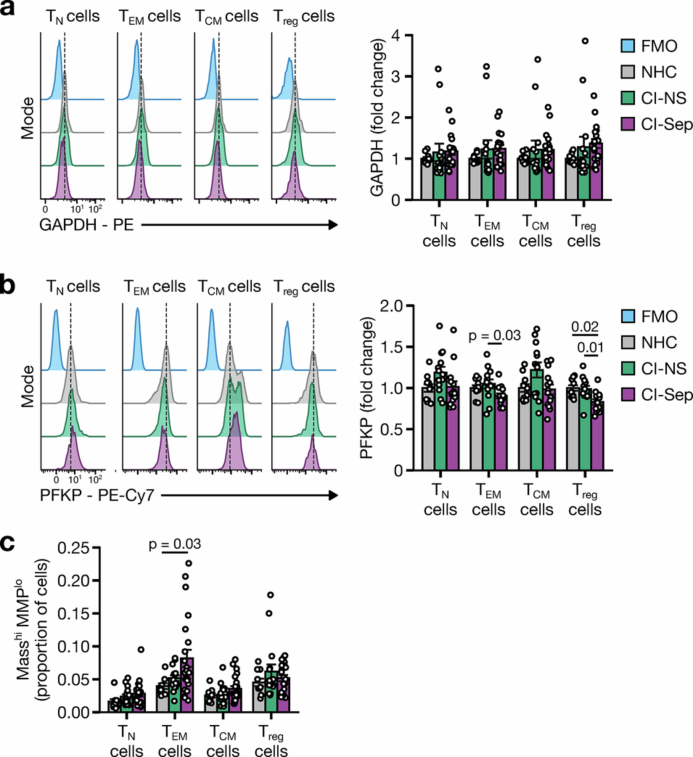
Additional glycolytic and mitochondrial characteristics of CD4^+^ T cell subsets in CI. **a**,**b**, Representative flow cytometry histograms (left) and quantification (right) of expression of glyceraldehyde 3-phosphate dehydrogenase (GAPDH) (**a**) and phosphofructokinase (PFKP) (**b**) in CD4^+^ T_N_, T_EM_, T_CM_, and T_reg_ cells from day 2 post-ICU admission. Shown as fold change of the MFI in CI-NS (GAPDH, *n* = 14; PFKP, *n* = 13) and CI-Sep (GAPDH, *n* = 19; PFKP, *n* = 14) relative to NHC (*n* = 11). **c**, Proportion of cells with high mitochondrial mass and low mitochondrial membrane potential (MMP) (Mass^hi^ MMP^lo^) among CD4^+^ T_N_, T_EM_, T_CM_, and T_reg_ cells from NHC (*n* = 10), CI-NS (*n* = 16), and CI-Sep (*n* = 22) at day 2 post-ICU admission. Statistical analysis for a-c was performed using the Kruskal–Wallis test followed by Dunn’s post-hoc testing. Data are presented as mean ± SEM. Each data point represents an individual donor. FMO = fluorescence minus one control; MFI = mean fluorescence intensity (geometric). Source data

Profiling of glycolytic and oxidative features of CD4^+^ T cell subsets indicated that glucose transporter 1 (GLUT1) and the glycolytic enzymes hexokinase 1 (HK1) and lactate dehydrogenase A (LDHA) were broadly increased in CI-NS and CI-Sep (Fig. 2a-c), whereas GAPDH was not substantially changed compared to the NHC CD4^+^ T cell subset counterparts (Extended Data Fig. 3a). Phosphofructokinase (PFKP) was modestly decreased in CI-Sep T_reg_ cells compared with NHC T_reg_ cells (Extended Data Fig. 3b). Additionally, the mitochondrial electron carrier cytochrome *c*, which is central to oxidative phosphorylation, was similarly expressed between CI and NHCs across all CD4^+^ T cell subsets (Fig. 2d). Analysis of mitochondrial attributes showed that CD4 T_N_ cells had higher mitochondrial mass, mitochondrial membrane potential (MMP) and ratio of MMP:mass in CI-Sep compared with NHCs (Fig. 2e-i). CI-Sep T_EM_ cells were enriched for a population of cells with high mitochondrial mass, but negligible MMP (Mass^hi^MMP^lo^) (Extended Data Fig. 3c), consistent with severe mitochondrial dysfunctional and/or cell death and aligning with the high rate of attrition of CD4^+^ T_EM_ cells in CI. Overall, CI drove broad metabolic remodeling across CD4^+^ T cell subsets, with T_reg_ cells showing the most pronounced glycolytic augmentation and resistance to attrition.

### T_reg_ cells retain suppressive features with stress in CI

**Fig. 3:**
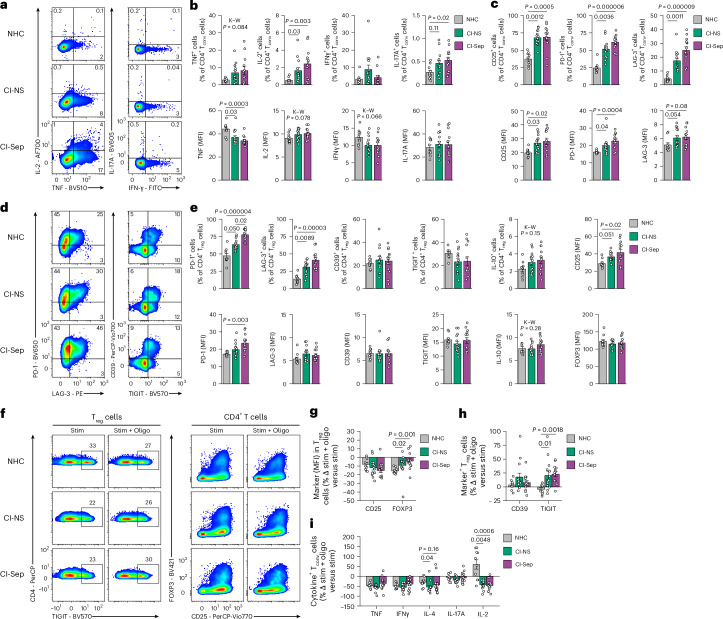
Mitochondrial stress triggers altered functionality in CD4^+^ T cells. **a**, Representative flow cytometry plots showing TNF, IL-2, IFNγ and IL-17A expression in NHCs, CI-NS and CI-Sep CD4^+^FOXP3^−^ T_conv_ cells from PBMCs isolated day 2 post-ICU admission and stimulated with CD3/CD28/CD2 antibodies for 24 h. **b**, Frequency of TNF^+^, IL-2^+^, IFNγ^+^ or IL-17A^+^ cells among T_conv_ cells (top) and the MFI of TNF, IL-2, IFNγ or IL-17A among T_conv_ cells positive for each cytokine (bottom) in NHCs (*n* = 10), CI-NS (*n* = 14), and CI-Sep (*n* = 13) CD4^+^FOXP3^−^ T_conv_ cells as in **a**. **c**, Frequency of CD25^+^, PD-1^+^ or LAG-3^+^ cells among T_conv_ cells (top) and the MFI of CD25, PD-1 or LAG-3 among T_conv_ cells positive for each marker (bottom) in NHCs (*n* = 10), CI-NS (*n* = 14) and CI-Sep (*n* = 13) CD4^+^FOXP3^−^ T_conv_ cells as in **a**. **d**, Representative flow cytometry plots showing PD-1, LAG-3, CD39 and TIGIT expression in NHC, CI-NS and CI-Sep CD4^+^FOXP3^+^ T_reg_ cells isolated and stimulated as in **a**. **e**, Frequency of PD-1^+^, LAG-3^+^, CD39^+^, TIGIT^+^ and IL-10^+^ cells among T_reg_ cells in NHCs (*n* = 10), CI-NS (*n* = 14) and CI-Sep (*n* = 13) (top) and the MFI of PD-1, LAG-3, CD39, TIGIT, IL-10, CD25 and FOXP3 among CD4^+^FOXP3^+^ T_reg_ cells positive for each marker (top and bottom) in NHC (*n* = 10), CI-NS (PD-1, LAG-3, CD39, TIGIT, CD25 and FOXP3, *n* = 14; IL-10, *n* = 13) and CI-Sep (*n* = 13) donors. **f**, Representative flow cytometry plots showing TIGIT expression in NHC, CI-NS and CI-Sep CD4^+^FOXP3^+^ T_reg_ cells or FOXP3 and CD25 expression in NHC, CI-NS and CI-Sep CD4^+^ T cells from PBMCs isolated on day 2 post-ICU admission and stimulated with CD3/CD28/CD2 antibodies for 24 h with or without mitochondrial impairment with low-dose oligomycin (10 nM). **g**, Percentage change in MFI among CD4^+^FOXP3^+^ T_reg_ cells positive for CD25 and FOXP3 from stimulated, oligomycin-treated PBMCs relative to stimulated, untreated PBMCs isolated from NHC (*n* = 10), CI-NS (*n* = 14) and CI-Sep (*n* = 13) donors and treated as in **f**. **h**, Percentage change in the frequency of CD39^+^ or TIGIT^+^ among CD4^+^FOXP3^+^ T_reg_ cells from stimulated, oligomycin-treated PBMCs relative to stimulated, untreated PBMCs isolated from NHC (*n* = 10), CI-NS (*n* = 14) and CI-Sep (*n* = 13) donors and treated as in **f**. **i**, Percentage change in the frequency of TNF^+^, IFNγ^+^, IL-4^+^, IL-17A^+^ or IL-2^+^ cells among CD4^+^FOXP3^−^ T_conv_ cells from stimulated, oligomycin-treated PBMCs relative to stimulated, untreated PBMCs isolated from NHC (*n* = 10), CI-NS (*n* = 14) and CI-Sep (*n* = 13) donors and treated as in **f**. Statistical analysis for **b**,**c**,**e**,**g**–**i** was performed using the Kruskal–Wallis (K–W) test followed by Dunn’s post hoc testing. Data are presented as mean ± s.e.m. Each data point represents an individual donor. Source data

**Extended Data Fig. 4:**
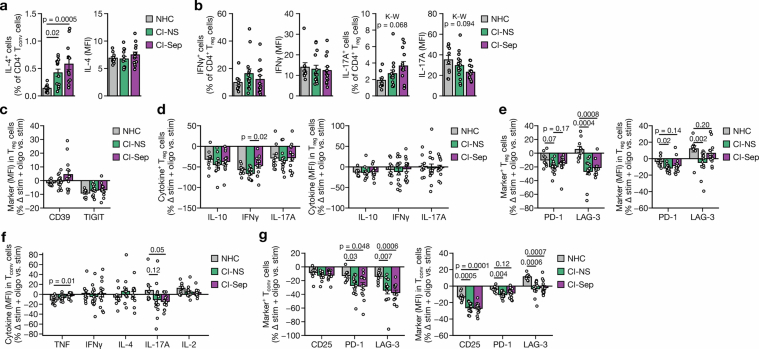
Additional functionalities of CD4^+^ T cells exposed to mitochondrial stress. **a**, Flow cytometric quantification of IL-4 expression in NHC (*n* = 10), CI-NS (*n* = 14), and CI-Sep (*n* = 13) CD4^+^FOXP3^−^ T_conv_ cells from PBMC isolated day 2 post-ICU admission stimulated with CD3/CD28/CD2 Ab for 24 h. Shown as the frequency of IL-4^+^ cells among T_conv_ cells (left) and the MFI of IL-4 among IL-4^+^ T_conv_ cells (right). **b**, Flow cytometric quantification of IFNγ and IL-17A expression as in (a) in NHC (*n* = 10), CI-NS (*n* = 14), and CI-Sep (*n* = 13) CD4^+^FOXP3^+^ T_reg_ cells. **c**, Flow cytometric quantification of CD39 and FOXP3 expression in NHC (*n* = 10), CI-NS (*n* = 14), and CI-Sep (*n* = 13) CD4^+^FOXP3^+^ T_reg_ cells from PBMC isolated day 2 post-ICU admission stimulated with CD3 + CD28 + CD2 Ab for 24 h with or without mitochondrial impairment with low-dose oligomycin (10 nM). Shown as the percent change in MFI among T_reg_ cells positive for each marker from stimulated, oligomycin-treated PBMC relative to stimulated, untreated PBMC. **d**, Flow cytometric quantification of IL-10, IFNγ, and IL-17A expression as in (c) in NHC (*n* = 10), CI-NS (IL-10^+^, IFNγ^+^, or IL-17A^+^ cells and IFNγ and IL-17A MFI, *n* = 14; IL-10 MFI, *n* = 13), and CI-Sep (*n* = 13) CD4^+^FOXP3^+^ T_reg_ cells. **e**, Flow cytometric quantification of PD-1 and LAG-3 expression as in (c) in NHC (*n* = 10), CI-NS (*n* = 14), and CI-Sep (*n* = 13) CD4^+^FOXP3^+^ T_reg_ cells. **f**, Flow cytometric quantification of TNF, IFNγ, IL-4, IL-17A, and IL-2 expression as in (c) in NHC (*n* = 10), CI-NS (*n* = 14), and CI-Sep (*n* = 13) CD4^+^FOXP3^−^ T_conv_ cells. **g**, Flow cytometric quantification of CD25, PD-1, and LAG-3 expression as in (c) in NHC (*n* = 10), CI-NS (*n* = 14), and CI-Sep (*n* = 13) CD4^+^FOXP3^−^ T_conv_ cells. Statistical analysis was performed using the Kruskal–Wallis test followed by Dunn’s post-hoc testing. Data are presented as mean ± SEM. Each data point represents an individual donor. K–W = Kruskal–Wallis; MFI = mean fluorescence intensity (geometric). Source data

To assess the functional capacities of CD4^+^FOXP3^−^ T_conv_ and CD4^+^FOXP3^+^T_reg_ cells in CI, PBMCs isolated from 10 NHCs, 14 CI-NS and 13 CI-Sep were stimulated with antibodies against CD3, CD28 plus CD2 (CD3/CD28/CD2 antibodies) to stimulate the T cell antigen receptor for 24 h and analyzed by flow cytometry. The frequency of TNF^+^, IL-17A^+^ and IL-4^+^CD4^+^ T_conv_ cells was higher, whereas the frequency of IFNγ^+^CD4^+^ T_conv_ cells was similar, in CI-Sep compared to NHCs (Fig. 3a,b and Extended Data Fig. 4a); however, the mean fluorescence intensity (MFI) of TNF among TNF^+^CD4^+^ T_conv_ cells was lower in CI-Sep compared with NHCs (Fig. 3b), consistent with an exhausted-like phenotype. IL-2^+^CD4^+^ T_conv_ cells were also more frequent in CI-NS and CI-Sep compared with healthy controls (Fig. 3b), potentially reflecting autocrine and/or paracrine survival signaling. CD4^+^ T_conv_ cells in CI-NS and CI-Sep exhibited increased frequency and MFI of activation (CD25) and checkpoint (PD-1 and LAG-3) proteins compared with CD4^+^ T_conv_ cells in NHCs (Fig. 3c), indicating a mixed phenotype of activation and impairment consistent with their cytokine profile.

T_reg_ cells also exhibited functional and phenotypic differences in CI-NS and CI-Sep compared with NHCs. CI-NS and CI-Sep had a higher frequency of PD-1^+^ and LAG-3^+^ T_reg_ cells, markers associated with suppressive effector T_reg_ cells in humans, compared with NHCs^22,23^ (Fig. 3d,e). In addition, CD39 and TIGIT are markers of highly suppressive T_reg_ cells that inhibit T_conv_ cells by several mechanisms including participating in the hydrolysis of proinflammatory extracellular ATP^24^ and by promoting secretion of (FGL2^25^, respectively. Both CD39 and TIGIT had sustained expression across NHC, CI-NS and CI-Sep T_reg_ cells compared with NHCs (Fig. 3d,e). The frequency and MFI of IL-10, as well as expression of proinflammatory cytokines like IFNγ and IL-17A were also comparable in NHC, CI-NS and CI-Sep T_reg_ cells (Fig. 3e and Extended Data Fig. 4b). CI-Sep and CI-NS T_reg_ cells had increased expression of CD25, which is associated with suppressive function^26^, and similar expression of the transcription factor FOXP3 compared with NHC T_reg_ cells (Fig. 3e). Together, these expression patterns point to an overall more suppressive phenotype of CI T_reg_ cells relative to NHC T_reg_ cells.

Increased metabolic plasticity in subsets of CD4^+^ T cells may enable adaptation to mitochondrial stress^27^, which is systemic in CI. To investigate this interplay, we stimulated PBMCs from 10 NHC, 14 CI-NS and 13 CI-Sep with or without a sublethal dose (10 nM) of the mitochondrial electron transport complex V inhibitor oligomycin for 24 h to partially impair mitochondrial energetics and simulate ICU-associated mitochondrial stress. Expression of FOXP3, which directly correlates with T_reg_ cell suppressor activity^28^, decreased in NHC T_reg_ cells (−16%), but was relatively preserved in CI-NS (−9%) and CI-Sep T_reg_ cells (−4%) incubated with oligomycin (Fig. 3f,g). The frequency of highly suppressive TIGIT^+^ T_reg_ cells increased in CI-NS (+21%) and CI-Sep (+23%) but not in NHC (−4%) when stimulated and exposed to oligomycin compared with stimulation alone, although TIGIT MFI in TIGIT^+^ T_reg_ cells was modestly and equivalently reduced in NHC, CI-NS and CI-Sep T_reg_ cells under the same conditions (Fig. 3f,h and Extended Data Fig. 4c). Expression of CD25, CD39 and IL-10 changed variably in T_reg_ cells incubated with oligomycin, but the changes did not differ between NHC, CI-NS and CI-Sep (Fig. 3g,h and Extended Data Fig. 4c,d). The frequency of LAG-3^+^ T_reg_ cells was reduced in CI-NS and CI-Sep, but not in NHC stimulated and oligomycin-incubated PBMCs, compared with stimulation alone (Extended Data Fig. 4e). Thus, the more metabolically plastic T_reg_ cells in CI had increased resistance to mitochondrial stress and maintained expression of FOXP3 and TIGIT.

The frequency of TNF^+^, IFNγ^+^, IL-4^+^ and IL-17A^+^ T_conv_ cells decreased to varying degrees in stimulated oligomycin-exposed PBMCs compared with stimulation alone, but the magnitude of reduction was comparable between NHC, CI-NS and CI-Sep (Fig. 3i). There were modest differences in the MFI of IL-17A and TNF in CD4 T_conv_ cells in CI-Sep versus NHCs after oligomycin treatment (Extended Data Fig. 4f). By contrast, expression of CD25, PD-1 and LAG-3 were more strongly reduced in oligomycin-treated CD4 T_conv_ cells in CI-NS and CI-Sep compared with NHCs (Extended Data Fig. 4g). Expression of the pro-survival cytokine IL-2 was impaired in oligomycin-treated CD4 T_conv_ cell from CI-NS and CI-Sep compared with NHCs (Fig. 3i), underscoring a metabolically enforced vulnerability that may predispose to attrition in CI. In summary, CI T_reg_ cells resisted mitochondrial stress and thereby maintained a suppressive phenotype, whereas CI T_conv_ exhibited persistent vulnerability to mitochondrial impairment with loss of effector function.

### CI globally remodels CD4^+^ T cell transcriptional states

**Fig. 4:**
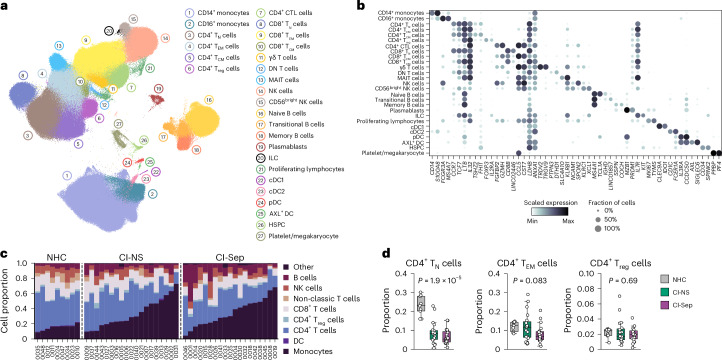
Single-cell transcriptomic map of CI. **a**, UMAP projection of 644,147 cells from isolated PBMCs of NHC (*n* = 9), CI-NS (*n* = 19) and CI-Sep (*n* = 19) donors collected day 2 post-ICU admission with cell lineage annotations. **b**, Dot plot showing canonical cell lineage-defining markers for the populations annotated in **a**. **c**, Proportionality analysis of major cell lineage families in NHCs (*n* = 9), CI-NS (*n* = 19) and CI-Sep (*n* = 19). Each column is an individual sample. **d**, Proportions of CD4^+^ T_N_, CD4^+^ T_EM_ and T_reg_ cell subsets in NHCs (*n* = 9), CI-NS (*n* = 19) and CI-Sep (*n* = 19). Statistical analysis for **d** was performed using the Empirical Bayes moderated analysis of variance (ANOVA) on logit-transformed proportions. Boxplots show the median with boxes bounded by IQR (25–75th percentile) and whiskers extending to the minimum and maximum values within 1.5 × IQR. Each data point represents an individual donor. CTL, cytotoxic T lymphocyte; DC, dendritic cell; DN, double negative; ILC, innate lymphoid cell; MAIT, mucosal-associated invariant T; NK, natural killer; HSPC, hematopoietic stem and progenitor cells. Source data

**Extended Data Fig. 5:**
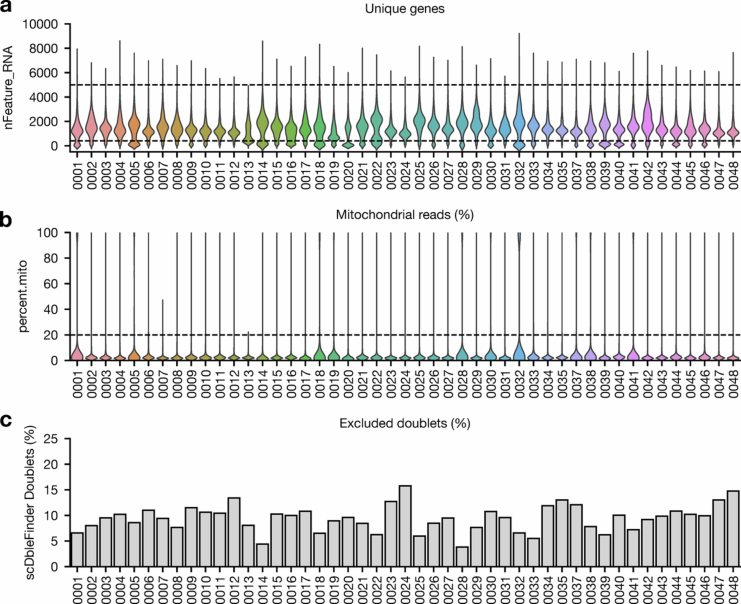
scRNA-seq quality control metrics. **a**, Number of unique genes (nFeature_RNA) per cell after artifact and ambient RNA removal with CellBender. Dashed lines represent subsequent filtering at 400 and 5000 features. **b**, Percentage of mitochondrial reads (percent.mito) per cell after artifact and ambient RNA removal with CellBender. Dashed line represents subsequent filtering threshold of 20% mitochondrial reads. **c**, Frequency of doublets removed with scDblFinder. Each sample is listed along the x-axis.

To investigate the mechanisms of CD4^+^ T cell metabolic remodeling, we performed single-cell RNA sequencing (scRNA-seq) on PBMCs from 9 NHC, 19 CI-NS and 19 CI-Sep donors. This yielded a dataset consisting of 644,147 cells, which were annotated using canonical transcriptional markers as 9 major cell lineages and 27 distinct cell populations (Fig. 4a,b and Extended Data Fig. 5). The proportion of total *CD14*^*+*^ and *CD16*^*+*^ monocytes was increased and that of *CD4*^+^ T lymphocytes was reduced in CI compared with NHCs (Fig. 4c). Among the *CD4*^+^ T cell subsets, we observed a reduction or tendency for reduction in the proportion of *CD4*^+^ T_N_ cells and *CD4*^+^ T_EM_ cells and a relative preservation of T_reg_ cell proportion in CI compared with healthy controls (Fig. 4d), broadly consistent with the flow cytometry data.

**Fig. 5:**
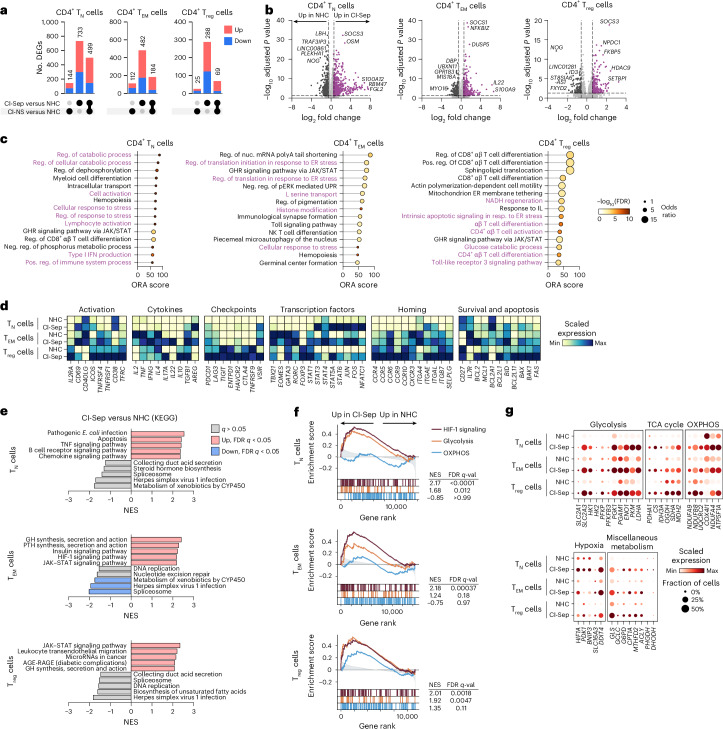
Transcriptomic features of CD4^+^ T cell subsets in CI. **a**, Differential gene expression by DESeq2 in pseudobulked scRNA-seq samples of NHC (*n* = 9), CI-NS (*n* = 19) or CI-Sep (*n* = 19) CD4^+^ T_N_, T_EM_ and T_reg_ cells. Comparisons reported for CI-NS relative to NHCs and CI-Sep relative to NHCs. Intersections shown via upset plot. **b**, Differential gene expression as in **a** shown as volcano plots for CD4^+^ T_N_ cells, T_EM_ cells and T_reg_ cells from CI-Sep (*n* = 19) versus NHCs (*n* = 9). Dashed lines denote a fold change >1.5 and a *P*_adj_ < 0.05. The top five up- and downregulated genes for each comparison are annotated. **c**, Over-representation analysis using the Gene Ontology Biological Processes (GO:BP) library among differentially expressed genes with *P*_adj_ < 0.05 as in **a** for CD4^+^ T_N_, T_EM_ and T_reg_ cells from CI-Sep (*n* = 19) versus NHCs (*n* = 9). The top 15 GO:BP pathways are shown, and those of interest related to T cell activation, stress and metabolism are denoted in purple. **d**, Heatmap showing single-cell normalized, log-transformed mean expression of representative genes associated with CD4^+^ T cell activation, cytokine production, immune checkpoints, transcription factors, homing and cell survival and apoptosis in CD4^+^ T_N_, T_EM_ and T_reg_ cells from NHCs (*n* = 9) and CI-Sep (*n* = 19). **e**, GSEA showing normalized enrichment scores (NES) for KEGG library pathways in CI-Sep (*n* = 19) versus NHC (*n* = 9) CD4^+^ T_N_, T_EM_ and T_reg_ cells. Gene ranks determined by DESeq2 as in **a**. The top five enriched and de-enriched gene sets by NES are shown. **f**, Selected KEGG library gene sets from **e** shown as running enrichment scores for CI-Sep (*n* = 19) versus NHC (*n* = 9) CD4^+^ T_N_, T_EM_ and T_reg_ cells. **g**, Dot plot showing single-cell normalized, log-transformed mean expression of selected metabolic and hypoxia-associated genes in NHC (*n* = 9) and CI-Sep (*n* = 19) CD4^+^ T_N_, T_EM_ and T_reg_ cells. Statistical analysis was performed using the Wald test and Benjamini–Hochberg false discovery rate (FDR) correction for multiple testing (**a**,**b**); a one-sided Fisher’s exact test with Benjamini–Hochberg FDR correction for multiple testing (**c**); and testing of the enrichment score (weighted Kolmogorov–Smirnov statistic) with Benjamini–Hochberg FDR correction for multiple testing (**e**,**f**). ORA, over-representation analysis.

Pseudobulked differential gene expression analysis with DESeq2 to evaluate sample-level differences indicated that most transcriptional changes were unique to CI-Sep, or shared between CI-NS and CI-Sep (Fig. 5a and Supplementary Table 1). Accordingly, we focused subsequent analyses on CI-Sep compared with NHCs. All CI-Sep *CD4*^+^ T cell subsets had increased expression of inflammatory regulators (*SOCS1* or *SOCS3*) among the top differentially expressed genes (Fig. 5b). CI-Sep *CD4*^+^ T_EM_ cells showed increased expression of activation-related (*NFKBIZ* and *IL22*) and negative feedback regulation (*DUSP5*) transcripts, whereas CI-Sep T_reg_ cells exhibited increased expression of a glucocorticoid-responsive transcript (*FKBP5*) (Fig. 5b). Over-representation analysis found enrichment of cellular stress pathways across all CI-Sep *CD4*^+^ T cell subsets compared with NHCs (Fig. 5c and Supplementary Table 2). CI-Sep *CD4*^+^ T_N_ cells showed transcriptional evidence of cellular activation (GOBP_LYMPOCYTE_ACTIVATION, GOBP_REGULATION_OF_CATABOLIC_PROCESS) (Fig. 5c), consistent with known bystander effects in sepsis^29,30^, whereas CI-Sep T_reg_ cells showed disproportionate enrichment of pathways related to glucose catabolism (GOBP_GLUCOSE_CATABOLIC_PROCESS) as well as NADH regeneration (GOBP_NADH_REGENERATION) (Fig. 5c), which supports cellular redox balance.

To further understand the transcriptional states of the *CD4*^+^ T cell subsets, we examined the expression patterns of individual genes canonically associated with core effector and regulatory programs in *CD4*^+^ T_N_, *CD4*^+^ T_EM_ and T_reg_ cells. Activation markers (*CD69*, *ICOS* and *TFRC*) were increased across all *CD4*^+^ T subsets in CI-Sep compared to NHCs (Fig. 5d). Some transcripts increased (*IFNG*, *IL17A* and *IL22*) and others decreased (*IL2* and *TNF*) in *CD4*^+^ T_EM_ cells in CI-Sep compared to NHCs (Fig. 5d). Mediators associated with wound healing and tissue repair (*TGFB1* and *AREG*) were increased in CI-Sep *CD4*^+^ T_N_ and T_EM_ cells compared to NHCs (Fig. 5d), suggesting a role in tissue homeostasis beyond antimicrobial defense. CI-Sep T_reg_ cells had increased expression of *IL10*, increased expression of transcripts encoding checkpoints markers (*CTLA4*, *PDCD1* and *LAG3*), and persistently high expression of *TIGIT* and *ENTPD1* compared to NHC T_reg_ cells (Fig. 5d). CI-Sep T_reg_ cells also showed comparable expression of *FOXP3* relative to NHC T_reg_ cells (Fig. 5d). *STAT5A* was increased in CI-Sep *CD4*^+^ T_N_ cells, *CD4*^+^ T_EM_ cells and T_reg_ cells compared to NHCs (Fig. 5d), likely reflecting enhanced IL-2 and IL-7 signaling tone. CI-Sep *CD4*^+^ T_EM_ cells and T_reg_ cells exhibited broadly remodeled chemokine and homing receptor expression (for example decreased *CD4*^+^ T_EM_ cell *CXCR3* and increased T_reg_ cell *CCR5* in CI-Sep relative to NHCs) (Fig. 5d), suggesting altered tissue recirculation. Finally, *CD4*^+^ T_N_, *CD4*^+^ T_EM_ and T_reg_ cells in CI-Sep expressed increased pro-apoptotic (*BAX* and *BAK1*) and anti-apoptotic (*BCL2* and *MCL2*) transcripts compared to the NHC counterparts (Fig. 5d), consistent with severe cellular stress and a compensatory survival program.

We orthogonally validated the over-representation analysis approach with gene set enrichment analysis (GSEA) using the Kyoto Encyclopedia of Genes and Genomes (KEGG) library, which broadly encompasses cellular processes and includes a robust group of metabolic gene sets. Very few pathways were significantly downregulated, limited to those related to spliceosome, herpes simplex virus 1 infection and metabolism of xenobiotics by CYP450 in CI-Sep *CD4*^+^ T_EM_ cells relative to NHC *CD4*^+^ T_EM_ cells (Fig. 5e and Supplementary Table 3). *CD4*^+^ T_N_ cells were marked by enrichment of pathways related to bacterial infection, apoptosis and inflammatory signaling (Fig. 5e). JAK-STAT signaling was significantly enriched in CI-Sep *CD4*^+^ T_EM_ and T_reg_ cells compared to NHCs, with additional enrichment of leukocyte transendothelial migration and microRNA pathways in CI-Sep T_reg_ cells compared to NHCs (Fig. 5e). All *CD4*^+^ T cell subsets showed significant enrichment of HIF-1 signaling in CI-Sep compared to NHCs (Fig. 5f), consistent with widespread cellular hypoxia, a context in which metabolic plasticity may be critical to sustain cellular fitness and function. Glycolysis was enriched in CI-Sep *CD4*^+^ T_N_ cells and T_reg_ cells with minimal change in *CD4*^+^ T_EM_ compared to their NHC counterparts (Fig. 5f). A trend toward oxidative phosphorylation enrichment was observed only in CI-Sep T_reg_ cells relative to NHC T_reg_ cells (Fig. 5f).

Evaluation of individual metabolic gene expression profiles in single cells indicated all CI-Sep *CD4*^+^ T cell subsets showed increased expression of genes involved in glucose transport (*SLC2A3*, encoding GLUT3), glycolytic processing (*HK1*, *PFKP* and *PKM*) and lactate production (*LDHA*) compared to NHCs (Fig. 5g). Tricarboxylic acid cycle and oxidative phosphorylation transcript abundance changes in CI-Sep *CD4*^+^ T_N_, *CD4*^+^ T_EM_ and T_reg_ cells were more modest and heterogenous compared to NHCs (Fig. 5g). Hypoxia-related genes (*HIF1A*, *SLC16A3* and *DDIT4*) were consistently higher across CI-Sep *CD4*^+^ T_N_, *CD4*^+^ T_EM_ and T_reg_ cells compared to NHCs, as were several other core metabolic regulators including in fatty acid oxidation (*CPT1A*) and one-carbon metabolism (*MTHFD2*) (Fig. 5g). No single gene seemed to mediate the *CD4*^+^ T cell subset-specific difference in glycolytic capacity, though several transcripts (such as *SLC2A3* and *PFKP*) were numerically most enriched in CI-Sep T_reg_ cells compared to CI-Sep *CD4*^+^ T_N_ and T_EM_ cells (Fig. 5g). Overall, CI and sepsis globally rewired *CD4*^+^ T cell transcriptional states toward cellular activation and functional impairments, inflammatory stress and metabolic adaptation, with the most pronounced enhancement of glycolytic features found in T_reg_ cells.

### Sepsis status subtly alters CD4^+^ T cell transcription

**Extended Data Fig. 6:**
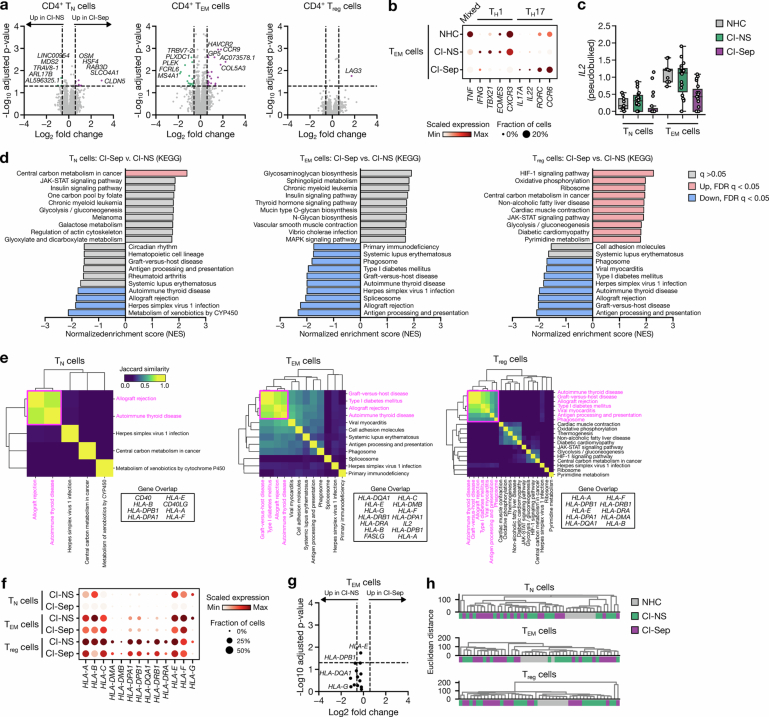
Transcriptomic comparison of CI-NS versus CI-Sep CD4^+^ T cells. **a**, Volcano plots showing differential gene expression by DESeq2 in pseudobulked scRNA-seq samples of CI-Sep (*n* = 19) versus CI-NS (*n* = 19) CD4^+^ T_N_, T_EM_, and T_reg_ cells. Dashed lines denote a fold change > 1.5 and a p_adj_ < 0.05. **b**, Dot plot showing single-cell normalized, log-transformed mean expression of selected CD4^+^ T_H_ genes in NHC (*n* = 9), CI-NS (*n* = 19), and CI-Sep (*n* = 19) CD4^+^ T_N_, T_EM_, and T_reg_ cells. **c**, Normalized scRNA-seq pseudobulked *IL2* expression in NHC (*n* = 9), CI-NS (*n* = 19, and CI-Sep (*n* = 19) CD4^+^ T_N_ and T_EM_ cells. **d**, GSEA showing the top enriched and de-enriched gene sets by normalized enrichment scores (NES) for KEGG pathways in CI-Sep (*n* = 19) versus CI-NS (*n* = 19) CD4^+^ T_N_, T_EM_, and T_reg_ cells. **e**, Hierarchical clustering and similarity matrix of KEGG pathways (p_adj_ < 0.05) as in (d) in CI-Sep (*n* = 19) versus CI-NS (*n* = 19) CD4^+^ T_N_, T_EM_, and T_reg_ cells based on Jaccard similarity index of their leading-edge genes. Clustered pathways of interest are annotated in pink with gene overlap reported. **f**, Dot plot showing single-cell normalized, log-transformed mean expression of HLA genes in CI-NS (*n* = 19) and CI-Sep (*n* = 19) CD4^+^ T_N_, T_EM_, and T_reg_ cells. **g**, Volcano plots showing differential gene expression as in (a) of HLA genes in CI-Sep (*n* = 19) versus CI-NS (*n* = 19) CD4^+^ T_EM_ cells. Dashed lines denote a fold change > 1.5 and a p_adj_ < 0.05. **h**, Hierarchical clustering of NHC (n = 9), CI-NS (n = 19), and CI-Sep (n = 19) CD4^+^ T_N_, T_EM_, and T_reg_ cells. Statistical analysis for a,g was performed using the Wald test and Benjamini–Hochberg FDR correction for multiple testing; for d using permutation testing of the enrichment score (weighted Kolmogorov–Smirnov statistic) with Benjamini–Hochberg FDR correction for multiple testing. Boxplots show the median with boxes bounded by interquartile range (IQR, 25–75th percentile) and whiskers extending to the minimum and maximum values within 1.5 x IQR. Each data point for c represents an individual donor.

Next, pseudobulked differential expression analysis of the scRNA-seq dataset revealed few genes with robust statistically significant differences between CI-Sep (*n* = 19 donors) and CI-NS (*n* = 19 donors), although immune checkpoint markers were increased in CI-Sep *CD4*^+^ T_EM_ (*HAVCR2*) and T_reg_ (*LAG3*) cells compared to CI-NS (Extended Data Fig. 6a and Supplementary Table 1). At single-cell level, CI-NS *CD4*^+^ T_EM_ cells had a tendency toward a T_H_1 cell gene expression profile (*IFNG*, *TBX21*, *EOMES* and *CXCR3*), whereas CI-Sep *CD4*^+^ T_EM_ cells had a T_H_17-biased signature (*IL17A*, *IL22*, *RORC* and *CCR6*) (Extended Data Fig. 6b). In addition, the number of *IL2* transcripts in *CD4*^+^ T_N_ and T_EM_ cells was reduced in CI-Sep compared to CI-NS (Extended Data Fig. 6c). To better explore these subtle differences, we performed GSEA using the KEGG pathway library. The most enriched pathways in CI-Sep relative to CI-NS, including HIF-1 signaling, oxidative phosphorylation and glycolysis, were restricted to T_reg_ cells (Extended Data Fig. 6d and Supplementary Table 3). By contrast, numerous pathways were downregulated across all *CD4*^+^ T cell subsets in CI-Sep compared to CI-NS, including autoinflammatory gene sets with overlapping leading-edge genes belonging to the HLA family (Extended Data Fig. 6d,e). HLA transcripts, which have been linked to CD4^+^ T cell effector cytokine production, proliferation and survival^31,32^, had a slight tendency for reduction in expression at the single-cell and sample level in CI-Sep *CD4*^+^ T cells compared to CI-NS *CD4*^+^ T cells (Extended Data Fig. 6f,g), aligning with reports of reduced HLA expression in T cells from trauma patients who subsequently develop sepsis^33^. Broadly, CI-NS and CI-Sep *CD4*^+^ T cells generally clustered together based on their largely shared transcriptome and remained distinct from NHCs (Extended Data Fig. 6h). Together, sepsis status exerted only a modest impact on the overall *CD4*^+^ T cell transcriptome in CI.

### Mitochondrial ROS spurs CI T_reg_ cell glycolytic capacity

**Fig. 6:**
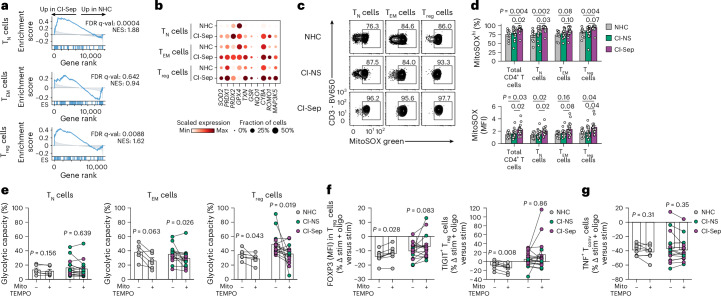
Mitochondrial ROS promotes CD4^+^ T_reg_ cell glycolytic acquisition in CI. **a**, GSEA showing running enrichment scores for ‘ROS pathway’ from the Hallmark library for CI-Sep (*n* = 19) versus NHC (*n* = 9) CD4^+^ T_N_, T_EM_ and T_reg_ cells. Gene ranks were determined by DESeq2 analysis of pseudobulked scRNA-seq transcriptomes. **b**, Dot plot showing single-cell normalized, log-transformed mean expression of selected ROS and antioxidant genes in CD4^+^ T_N_, T_EM_ and T_reg_ cells from NHC (*n* = 9) and CI-Sep (*n* = 19). **c**,**d** Representative flow cytometry plots (**c**) and quantification of mitochondrial ROS (**d**) determined by MitoSOX Green staining in CD4^+^ T_N_, T_EM_ and T_reg_ cells isolated on day 2 post-ICU admission from NHC (*n* = 9), CI-NS (*n* = 18) and CI-Sep (*n* = 19). MitoSOX^hi^ denotes the cells in the gated region. Percentages are of the CD4^+^ T cell subset parent gate. MitoSOX MFI shows the per-cell intensity of MitoSOX staining across all cells in each CD4^+^ T cell subset. **e**, Glycolytic capacity by puromycin-incorporation assay in NHC (*n* = 6), CI-NS (*n* = 7) and CI-Sep (*n* = 8) CD4^+^ T_N_, T_EM_ and T_reg_ cells from paired PBMC cultures treated with vehicle control or MitoTEMPO (10 μM) for 24 h. Displayed as percentage of the maximum puromycin incorporation without metabolic inhibitors. **f**, Percentage change in FOXP3 MFI among CD4^+^FOXP3^+^ T_reg_ cells (left) and percentage change in the frequency of TIGIT^+^ cells among CD4^+^FOXP3^+^ T_reg_ cells (right) from PBMCs stimulated with CD3/CD28/CD2 antibodies for 24 h and low-dose oligomycin (10 nM) with or without MitoTEMPO relative to CD3/CD28/CD2 antibody-stimulated, oligomycin-untreated, without MitoTEMPO, PBMCs from NHC (*n* = 8), CI-NS (*n* = 6) and CI-Sep (*n* = 10). **g**, Percentage change in the frequency of TNF^+^ cells among CD4^+^FOXP3^−^ T_conv_ cells from stimulated, oligomycin-treated PBMCs with vehicle or MitoTEMPO relative to stimulated, untreated PBMCs isolated from NHC (*n* = 8), CI-NS (*n* = 6) and CI-Sep (*n* = 10) donors and treated as in **f**. Statistical analysis was performed using permutation testing of the enrichment score (weighted Kolmogorov–Smirnov statistic) with Benjamini–Hochberg FDR correction for multiple testing (**a**); using the Kruskal–Wallis test with Dunn’s post hoc testing (**d**); and using paired sample testing with the two-sided Wilcoxon signed-rank test (**e**–**g**). Data for **d–g** are presented as mean ± s.e.m. Each data point represents an individual donor. Source data

Mitochondrial oxidative stress leads to the accumulation of mitochondrial ROS, which can promote HIF-1α and increase the expression of glycolytic genes^34^. GSEA of the scRNA-seq dataset identified significantly increased ROS pathway activity in *CD4*^+^ T_N_ cells and T_reg_ cells from CI-Sep relative to NHCs (Fig. 6a and Supplementary Table 3), indicating mitochondrial ROS could be a driver of glycolytic remodeling in CI. Single-cell analysis indicated many (*SOD2*, *TXN*, *MAP3K5* and *ROMO1*), although not all (*GPX4*), canonical ROS-associated genes were broadly increased in CI-Sep *CD4*^+^ T cells compared to NHCs (Fig. 6b). Flow cytometry showed increased mitochondrial ROS in CI-Sep CD4^+^ T cells compared to NHCs (Fig. 6c,d), whereas treatment of NHC (*n* = 6), CI-NS (*n* = 7) and CI-Sep (*n* = 8) PBMCs with MitoTEMPO, a mitochondrially targeted superoxide scavenger, for 24 h caused a large attenuation of the glycolytic remodeling in CI T_reg_ cells (pooled from CI-NS and CI-Sep) compared to vehicle-treated CI T_reg_ cells (mean glycolytic capacity 36% versus 49%, respectively) (Fig. 6e). A similar effect with MitoTEMPO treatment was not observed in CI CD4^+^ T_N_ cells, and only modestly detected in CI CD4 T_EM_ cells, compared to vehicle-treated CI T_N_ and T_EM_ cells, respectively (Fig. 6e). No appreciable effects of 24 h of MitoTEMPO treatment compared to vehicle treatment were observed on FOXP3 stabilization and TIGIT upregulation in CI T_reg_ cells or on expression of TNF in CI CD4^+^ T_conv_ cells in stimulated PBMCs exposed to mitochondrial impairment with low-dose oligomycin (Fig. 6f,g). These findings implicated mitochondrial ROS as a mechanistic contributor to the acquisition of glycolytic capacity but not regulation of FOXP3 or TIGIT expression in T_reg_ cells from patients who are CI.

### Kynurenine metabolism supports suppressive CI T_reg_ cells

**Fig. 7:**
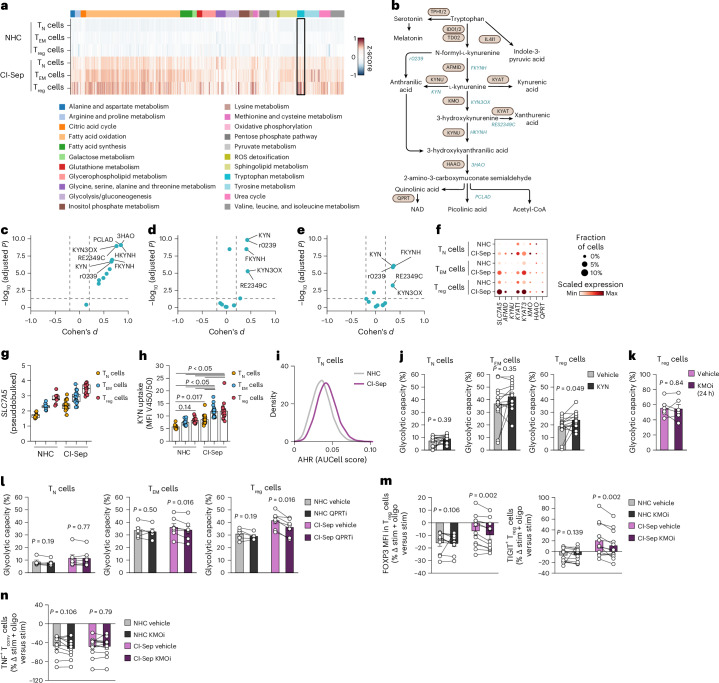
Kynurenine metabolism promotes CD4^+^ T_reg_ suppressive remodeling in CI patients. **a**, FBA as a heatmap showing in silico flux potential for individual metabolic reactions scaled by standard *z*-scoring in scRNA-seq data from NHCs and CI-Sep CD4^+^ T_N_, T_EM_ and T_reg_ cells. Larger values indicate higher predicted flux. **b**, Schematic of tryptophan and kynurenine metabolism showing metabolites (black), enzymes (tan) and Recon2 pathway annotations (blue). Panel **b** created in BioRender. Stier, M. (2025) https://BioRender.com/q07s159. **c**–**e**, FBA pairwise comparison of effect size of tryptophan metabolism reactions for CI-Sep T_reg_ cells versus NHC T_reg_ cells (**c**), CI-Sep T_reg_ cells versus CI-Sep CD4^+^ T_N_ cells (**d**) and CI-Sep T_reg_ cells versus CI-Sep CD4^+^ T_EM_ cells (**e**). Effect size reported as Cohen’s *d* value (standardized mean difference). **f**, Dot plot of single-cell normalized, log-transformed counts of kynurenine metabolism genes in NHC (*n* = 9) and CI-Sep (*n* = 19) CD4^+^ T_N_, T_EM_ and T_reg_ cells. Values are scaled within each gene. **g**, Normalized (log(CPM + 1)) scRNA-seq pseudobulked *SLC7A5* expression in NHC (*n* = 9) and CI-Sep (*n* = 19) CD4^+^ T_N_, T_EM_ and T_reg_ cells. CPM, counts per million. **h**, Flow cytometric detection of kynurenine uptake in NHC (*n* = 12) and CI-Sep (*n* = 18) CD4^+^ T_N_, T_EM_, and T_reg_ cells following a 5-min pulse with l-kynurenine (100 μM). **i**, AUCell pathway activity analysis of scRNA-seq data for a consensus human pan-tissue AHR gene signature in CI-Sep T_reg_ cells compared to NHC T_reg_ cells (Cohen’s *d* = 0.522, *P* = 6.44 × 10^−111^). Larger values represent higher AHR-associated transcriptional activity. **j**, Glycolytic capacity by puromycin-incorporation assay in NHC (*n* = 14) CD4^+^ T_N_, T_EM_ and T_reg_ cells from paired PBMC cultures treated with vehicle or l-kynurenine (KYN; 10 μM) for 24 h displayed as percentage of the maximum puromycin incorporation without metabolic inhibitors. **k**, Glycolytic capacity by puromycin-incorporation assay in CI-Sep (*n* = 6) T_reg_ cells from paired PBMC cultures treated with vehicle or KMO inhibitor (KMOi) GSK180 (10 μM) for 24 h displayed as percentage of the maximum puromycin incorporation without metabolic inhibitors. **l**, Glycolytic capacity by puromycin-incorporation assay in NHC (*n* = 5) T_N_, T_EM_ and T_reg_ cells and CI-Sep (*n* = 7) CD4^+^ T_N_, T_EM_ and T_reg_ cells from paired PBMC cultures treated with vehicle or QPRT inhibitor (QPRTi) phthalic acid (100 μM) for 24 h displayed as percentage of the maximum puromycin incorporation without metabolic inhibitors. **m**, Percentage change in FOXP3 MFI among CD4^+^FOXP3^+^ T_reg_ cells (left) or the percentage change in the frequency of TIGIT^+^ cells among CD4^+^FOXP3^+^ T_reg_ cells (right) from PBMCs isolated from NHC (*n* = 10) and CI-Sep (*n* = 12) at day 2 post-ICU admission, stimulated with CD3/CD28/CD2 antibodies for 24 h, treated with low-dose oligomycin (10 nM) and with vehicle or GSK180 (10 μM) relative to stimulated, untreated PBMCs. **n**, Percentage change in the frequency of TNF^+^ cells among T_conv_ cells from NHC (*n* = 10) and CI-Sep (*n* = 12) donors, stimulated, oligomycin-treated PBMCs with vehicle or KMO inhibitor GSK180 relative to stimulated, untreated PBMCs as in **m**. Statistical analysis was performed using a two-sided Mann–Whitney *U*-test with Benjamini–Hochberg FDR correction for multiple testing (**c**–**e**); a Kruskal–Wallis test followed by Dunn’s post hoc testing (**h**); a two-sided Wilcoxon signed-rank test for paired sample analysis (**j**,**k**,**m**,**n**); and a two-tailed permutation test for paired samples (**l**). Data for **h**,**j**–**n** are presented as mean ± s.e.m. Boxplots show the median with boxes bounded by IQR (25–75th percentile) and whiskers extending to the minimum and maximum values within 1.5 × IQR. Each data point represents an individual donor. Source data

**Extended Data Fig. 7:**
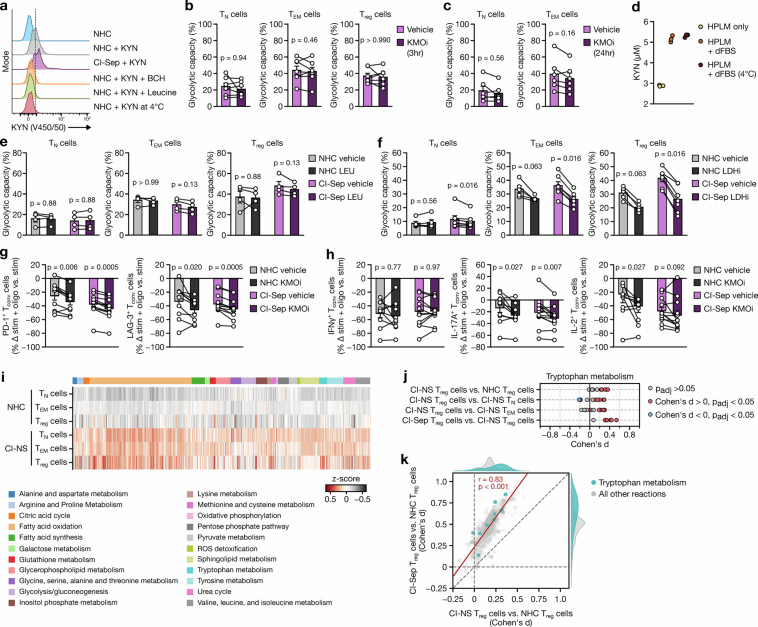
Additional effects of kynurenine on CD4^+^ T cells from CI-Sep patients. **a**, Flow cytometry histograms of kynurenine (KYN) uptake following 5 minute pulse of NHC and CI-Sep CD4^+^ T_reg_ cells with kynurenine (100 μM), without pre-treatment or pretreated with SLC7A5 inhibitor BCH (10 mM) or L-leucine (LEU, 5 mM) or incubated at 4 °C prior to adding kynurenine. **b**,**c**, Glycolytic capacity by puromycin-incorporation assay in CI-Sep CD4^+^ T_N_, T_EM_, and T_reg_ cells from paired PBMC cultures treated with vehicle or KMO inhibitor GSK180 (KMOi, 10 μM) for 3 h (*n* = 7) (b) or 24 h (*n* = 6) (c). Displayed as percentage of the maximum puromycin incorporation without metabolic inhibitors. **d**, LC–MS/MS showing kynurenine concentration in HPLM with or without dialyzed FBS (dFBS), fresh or stored at 4 °C for 30 days. **e**, Glycolytic capacity as in (b) in NHC (*n* = 4) and CI-Sep (*n* = 4) CD4^+^ T_N_, T_EM_, and T_reg_ cells from paired PBMC cultures treated with vehicle or L-Leucine (500 μM) for 24 h. **f**, Glycolytic capacity as in (b) in NHC (*n* = 5) and CI-Sep (*n* = 7) CD4^+^ T_N_, T_EM_, and T_reg_ cells from paired PBMC cultures treated with vehicle or LDHA inhibitor FX-11 (LDHi, 20 μM) for 24 h. **g**,**h** Flow cytometric quantification of PD-1 and LAG-3 expression (g) or IFNγ, IL-17A, and IL-2 cytokine expression (h) in NHC (*n* = 10) and CI-Sep (*n* = 12) CD4^+^FOXP3^−^ T_conv_ cells from PBMC isolated 2 days post-ICU admission stimulated with CD3/CD28/CD2 Ab for 24 h with or without low-dose oligomycin (10 nM) and treated with vehicle or KMO inhibitor GSK180 (10 μM). Shown as percent change in the frequency of PD-1^+^, LAG-3^+^, IFNγ^+^, IL-17A^+^, or IL-2^+^ cells among T_conv_ cells from stimulated, oligomycin-treated PBMC with vehicle or KMO inhibitor GSK180 relative to stimulated, untreated PBMC. **i**, FBA as a heatmap showing *in silico* flux potential (z-scored) for individual metabolic reactions in scRNA-seq data from NHC and CI-NS CD4^+^ T_N_, T_EM_, and T_reg_ cells. **j**, FBA pairwise comparisons of Cohen’s d effect size of tryptophan metabolism reactions among NHC, CI-NS, and CI-Sep T_N_, T_EM_, and T_reg_ cells. **k**, Scatterplot showing FBA results as in (i) for CI-NS T_reg_ cells versus NHC T_reg_ cells and CI-Sep T_reg_ cells versus NHC T_reg_ cells reported as Cohen’s d values. A linear regression line modeling the association between all reactions is shown in red. Statistical analysis for b,c,g,h was performed using with two-sided Wilcoxon signed-rank test for paired sample analysis; for e,f, using two-tailed permutation testing for paired samples; for j, using two-tailed Mann–Whitney U testing with Benjamini–Hochberg FDR correction; for k, using a two-sided t-test of the regression slope. Data for b,c,e-h are presented as mean ± SEM. Each data point in b,c,e-h represents a biologic or in d represents a technical replicate. Source data

To evaluate the factors that contributed to the stabilization of the suppressive phenotype, such as FOXP3 and TIGIT expression in CI-Sep T_reg_ cells, we used computational flux balance analysis (FBA). Across a curated set of 22 metabolic reaction families from the global human metabolism reconstruction (Recon2) encompassing pathways for major macronutrient metabolism and those relevant directly to T cells, CI-Sep *CD4*^+^ T_reg_ cells showed disproportionate enrichment in predicted flux through several pathways, including tryptophan metabolism, methionine and cysteine metabolism, glutathione metabolism, urea cycle and valine, leucine and isoleucine metabolism compared to CI-Sep T_N_ and T_EM_ and all NHC *CD4*^+^ T cell subsets (Fig. 7a and Supplementary Table 4). Tryptophan is metabolized by IDO1/2 or TDO2 into *N*-formyl-l-kynurenine, which is hydrolyzed to kynurenine (Fig. 7b). CD4^+^ T cells do not express IDO/TDO enzymes, but can uptake *N*-formyl-l-kynurenine and kynurenine through LAT1 (encoded by *SLC7A5*)^35^, which contribute to de novo nicotinamide adenine dinucleotide (NAD) synthesis, acetyl-CoA production and a variety of immunomodulatory intermediates^36–40^, including some that can activate the aryl hydrocarbon receptor (AHR) and/or promote T_reg_ cell suppressive capacity in healthy human T_reg_ cells^41,42^. Increased plasma levels of kynurenine are associated with increased morbidity and mortality in patients with sepsis^43,44^. Pairwise comparisons of FBA results showed that across CI-Sep T_reg_ cells versus NHC T_reg_ cells, CI-Sep *CD4*^+^ T_N_ cells or CI-Sep *CD4*^+^ T_EM_ cells, kynurenine-associated reactions were among the most substantially altered in the tryptophan metabolic network (Fig. 7c–e). There was a tendency for increased expression of genes for *N*-formyl-l-kynurenine and kynurenine import (*SLC7A5*), *N*-formyl-l-kynurenine hydrolysis (*AFMID*), kynurenine transamination (*KYAT1* and *KYAT3*) and quinolinic acid catabolism for de novo NAD synthesis (*QPRT*) in CI-Sep T_reg_ cells compared to NHC T_reg_ cells, CI-Sep *CD4*^+^ T_N_ cells or CI-Sep *CD4*^+^ T_EM_ cells (Fig. 7f,g). Based on the intrinsic fluorescent properties of kynurenine, we measured kynurenine uptake in CD4^+^ T cell subsets by flow cytometry^35^. LAT1-mediated kynurenine uptake was increased in all CI-Sep CD4^+^ T cell subsets, including CI-Sep T_reg_ cells, compared to NHC (Fig. 7h and Extended Data Fig. 7a). Consistent with this, CI-Sep T_reg_ cells exhibited an increased transcriptional signature of AHR activity compared to NHC T_reg_ cells^45^ (Fig. 7i).

To determine whether kynurenine exposure alone was sufficient to augment T_reg_ cell glycolytic capacity, we treated NHC PBMCs for 24 h with 10 μM kynurenine, a dose within the plasma concentration range in sepsis^43^. Exogenous kynurenine modestly but consistently increased the glycolytic capacity of NHC T_reg_ cells, an effect not observed in CD4^+^ T_N_ or T_EM_ cells (Fig. 7j). Conversely, short (3 h) or extended (24 h) treatment of CI-Sep PBMCs with the kynurenine-3-monooxygenase (KMO) inhibitor GSK180 did not alter the glycolytic capacity in any CD4^+^ T cell subset (Fig. 7k and Extended Data Fig. 7b,c), suggesting that central kynurenine intermediates were not contributing directly to the glycolytic capacity. This neutral impact was not due to a deficit of kynurenine in human plasma-like medium (HPLM), which we measured at near physiological levels (Extended Data Fig. 7d). In contrast to kynurenine, supplementation of NHC and CI-Sep PBMCs for 24 h with leucine, another LAT1 substrate, did not alter the glycolytic capacity of CD4^+^ T cell subsets including CD4^+^ T_reg_ cells, suggesting that the effect of kynurenine on T_reg_ cell glycolytic capacity is not a general property of all LAT1 transported amino acids (Extended Data Fig. 7e). Kynurenine metabolism can bypass KMO through anthranilic acid, which feeds further downstream reactions, including de novo NAD synthesis that could influence glycolysis (Fig. 7b). Treatment of NHC and CI-Sep PBMCs with the QPRT inhibitor phthalic acid for 24 h resulted in a modest reduction in glycolytic capacity in CI-Sep T_reg_ cells compared to vehicle-treated CI-Sep T_reg_ cells (Fig. 7l). As a control, inhibition of LDHA, a canonical enzyme in NAD/NADH recycling that sustains glycolysis, in NHC and CI-Sep PBMCs for 24 h produced a large reduction in glycolytic capacity broadly in CD4^+^ T_N_, T_EM_ and T_reg_ cells from NHCs and CI-Sep (Extended Data Fig. 7f). These observations suggested that while kynurenine metabolism was altered disproportionately in CI-Sep T_reg_ cells compared to CI-Sep T_N_ and T_EM_ cells, it had only a minimal role in the glycolytic reprogramming of CI-Sep T_reg_ cells, potentially via de novo NAD generation.

Kynurenine metabolism promotes the formation of suppressive T_reg_ cells in healthy individuals^41,42^, but direct evidence of its role in human systemic inflammatory diseases is limited. To test the role of kynurenine in sepsis, we stimulated NHC and CI-Sep PBMCs with CD3/CD28/CD2 antibodies for 24 h with low-dose (10 nM) oligomycin in the presence or absence of KMO inhibitor GSK180. Compared to vehicle-treated CI-Sep T_reg_ cells, treatment with GSK180 led to partial reversal of both FOXP3 stabilization (−6% with vehicle versus −10% with GSK180) and augmented TIGIT expression (20% with vehicle versus 10% with GSK180) in CI-Sep T_reg_ cells (Fig. 7m). No effect on FOXP3 or TIGIT expression was seen in NHC T_reg_ cells treated with GSK180 compared to NHC T_reg_ cells treated with vehicle (Fig. 7m). GSK180 further reduced the frequency of PD-1^+^ and LAG-3^+^CD4^+^ T_conv_ cells in both NHC and CI-Sep with GSK180 treatment compared to vehicle (Extended Data Fig. 7g), and had no effect on the frequency of CI-Sep TNF^+^, IFNγ^+^ or IL-2^+^ CD4^+^ T_conv_ cells, though GSK180 treatment led to a modestly greater reduction in the frequency of CI-Sep IL-17A^+^CD4^+^ T_conv_ cells compared to vehicle treatment (Fig. 7n and Extended Data Fig. 7h). Thus, impairment of kynurenine metabolism at the level of KMO selectively attenuated the suppressive features in CI-Sep T_reg_ cells without substantially compromising or exaggerating CI-Sep CD4^+^ T_conv_ cell effector function, highlighting a potential axis for rebalancing immune suppression and effector responses in sepsis.

We also applied FBA to CD4^+^ T cell subsets from CI-NS patients and found that the flux changes observed in CI-Sep CD4^+^ T cells were broadly conserved across the same major 22 Recon2 reaction families in CI-NS CD4^+^ T cells (Extended Data Fig. 7i and Supplementary Table 4). CI-NS T_reg_ cells had higher tryptophan metabolic flux than NHC T_reg_ cells, CI-NS CD4^+^ T_N_ or T_EM_ cells, though CI-NS T_reg_ cell tryptophan metabolic flux was less than CI-Sep T_reg_ cells (Extended Data Fig. 7j). Comparison of global effect sizes of metabolic flux across all reactions indicated a strong correlation between CI-Sep versus NHC and CI-NS versus NHC T_reg_ cells, with consistently larger effect sizes in CI-Sep T_reg_ cells (Extended Data Fig. 7k). Collectively, these data show that enhanced kynurenine metabolism promotes the stabilized suppressive phenotype of CI T_reg_ cells.

### T_reg_ cell glycolytic capacity links to poor ICU metrics

**Fig. 8:**
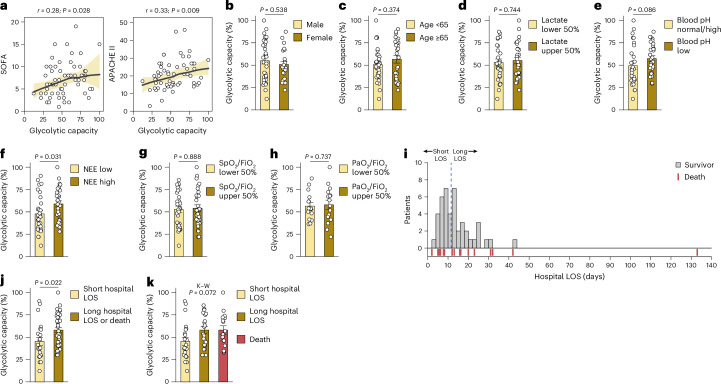
Association of CI T_reg_ cell glycolytic capacity with ICU clinical metrics. **a**, Spearman rank correlation showing the association between pooled CI T_reg_ cell (*n* = 60; 50% CI-Sep) glycolytic capacity and SOFA (left) and APACHE II scores (right). A locally weighted scatter-plot smoothing (LOWESS) curve was fitted and bootstrapped 95% confidence interval are shaded. **b**, Glycolytic capacity of T_reg_ cells from CI donors (*n* = 60) dichotomized by sex. **c**, Glycolytic capacity of T_reg_ cells from CI donors (*n* = 60) dichotomized by age <65 years (median 55 years, IQR 47–60) or ≥65 years (median 71 years, IQR 68–78). **d**, Glycolytic capacity of T_reg_ cells from CI donors (*n* = 58) by plasma lactate levels grouped as lower 50% (median 1.6 mmol l^−1^, IQR 1.1–1.8 and upper 50%, median 4.3 mmol l^−1^, IQR 3.1–5.8). **e**, Glycolytic capacity of T_reg_ cells from CI donors (*n* = 60) by plasma pH grouped as normal or alkalotic (median 7.40, IQR 7.37–7.44) versus low pH (median 7.26, IQR 7.20–7.29). **f**, Glycolytic capacity of T_reg_ cells from CI donors (*n* = 60) grouped by vasopressor dose, reported as norepinephrine equivalent dose (NEE), into low NEE (lower 50%, median 0.000 μg kg^−1^ min^−1^, IQR 0.000–0.036) and high NEE (upper 50%, median 0.197 μg kg^−1^ min^−1^, IQR 0.134–0.389). **g**, Glycolytic capacity of T_reg_ cells from CI donors (*n* = 60) by SpO_2_:FiO2 (S:F) ratio grouped into low S:F (lower 50%, median 136, IQR 110–176) and high S:F (upper 50%, median 338, IQR 262–433). **h**, Glycolytic capacity of T_reg_ cells from CI donors (*n* = 60) by PaO_2_:FiO_2_ (P:F) ratio grouped into low P:F (lower 50%, median 124, IQR 111–146) and high P:F (upper 50%, median 338, IQR 270–382). **i**, Histogram showing the hospital length of stay (LOS) in days among survivors to hospital discharge. The cohort median shown at 11.5 days (blue dashed line) was used to stratify into the shortest 50% (median 7 days, IQR 6–8) and longest 50% (median 16 days, IQR 13–23). Rug plot aligned below the histogram depicts the date of death post-admission for nonsurvivors. **j**, Glycolytic capacity of T_reg_ cells from CI donors (*n* = 60) grouped into short hospital LOS (*n* = 22) versus long hospital LOS or death (*n* = 38). **k**, Glycolytic capacity of T_reg_ cells from CI donors (*n* = 60) with short hospital LOS (*n* = 22), long hospital LOS (*n* = 22), or death (*n* = 16). Lactate measurement in **d** and arterial blood gas measurement of PaO_2_ in **h** was not performed by the treating clinical team in all patients; values shown where available. Statistical analysis was performed using a two-sided Mann–Whitney *U*-test (**b**–**h**) and a Kruskal–Wallis test (**k**). Data for **b–h**,**j**,**k** are presented as mean ± s.e.m.

Next, we analyzed the associations between T_reg_ cell glycolytic capacity and ICU metrics of overall disease severity, individual organ failures and clinical outcomes in pooled CI-NS (*n* = 30) and CI-Sep (*n* = 30) samples to gain adequate power. Glycolytic capacity in CI T_reg_ cells positively correlated with illness severity, including Sequential Organ Failure Assessment (SOFA) and Acute Physiology and Chronic Health Evaluation II (APACHE II) scores (Fig. 8a). No associations of CI T_reg_ cell glycolytic capacity were seen with sex or age (Fig. 8b,c). While CI T_reg_ cell glycolytic capacity was not correlated with peak plasma lactate, there was a trend between lower blood pH and higher T_reg_ cell glycolytic capacity (Fig. 8d,e). Patients with higher vasopressor requirements (with all vasopressor doses converted to a standardized norepinephrine equivalent dose), which is an indicator of more severe shock, exhibited higher T_reg_ cell glycolytic capacity compared to patients on lower or no vasopressors (Fig. 8f). There was no association between CI T_reg_ cell glycolytic capacity and severity of lung injury as measured by alveolar gas-exchange metrics (SpO_2_/FiO_2_ or PaO_2_/FiO_2_) (Fig. 8g,h).

Primary illness severity and the acquisition of secondary complications can prolong hospital length of stay or lead to in-hospital mortality. In this CI cohort, 16 individuals died during the hospitalization and 44 survived to hospital discharge. We grouped the 44 survivors into cohorts of short and long hospital lengths of stay (defined as below and above the median hospital length of stay, respectively) (Fig. 8i). Patients with a long hospital length of stay or in-hospital death had significantly higher T_reg_ cell glycolytic capacity than those with a short hospital length of stay (Fig. 8j), with similar effects observed when long length of stay and in-hospital death were examined individually (Fig. 8k). Together, these results linked CI T_reg_ cell metabolic remodeling to greater illness severity, more profound shock and acidemia, prolonged hospital length of stay and mortality in CI.

## Discussion

Here, we found that CI CD4^+^ T cells underwent subset-specific metabolic rewiring characterized by persistence of T_reg_ cells that showed enhanced glycolytic capacity and stabilization of suppressive features and attrition of less metabolically plastic CD4^+^ T_conv_ cells that showed functional impairments. Parallel changes in mitochondrial ROS and kynurenine metabolism in T_reg_ cells promoted glycolytic remodeling and suppressive adaptations, respectively. The magnitude of T_reg_ cell metabolic reprogramming was associated with greater illness severity and adverse clinical outcomes.

Our findings highlighted the need to model disease-specific factors, like metabolic stress, which was evident transcriptomically with robust hypoxic and cellular stress signatures across all CI CD4^+^ T cell. The association between adaptive glycolytic remodeling in CI T_reg_ cells and severe shock, but not impaired gas exchange in the lungs, suggested that these stress features may be imprinted from T cell transit through inadequately perfused, dysoxic tissues rather than arising from intrinsic respiratory failure. By modeling aspects of this physiological context through partial mitochondrial impairment ex vivo, we unmasked key CI T_reg_ cell phenotypes, including stabilization of FOXP3 and upregulation of TIGIT.

Kynurenine metabolism promoted immunosuppressive features in CI T_reg_ cells. While its effects were partial (reflecting the complex physiological changes in sepsis) our findings harmonize with previous clinical studies that linked elevated plasma kynurenine to poor clinical outcomes^43,44^ and extend previous findings, showing that kynurenine promotes T_reg_ cell programs in healthy human T cells^41,42^, to human inflammatory disease. Tryptophan and kynurenine metabolism are druggable^46^, and our data suggest KMO inhibition may support the restoration of immune balance by attenuating excessive T_reg_ cell immunosuppressive features without inducing CD4^+^ T_conv_ cell hyperactivation. In contrast to other candidate immunomodulatory strategies, kynurenine has both immune and nonimmune effects. Kynurenine is an arterial vasodilator^47^ and kynurenine-derived metabolites are both neurotoxic^48^ and tissue toxic^49,50^, which may contribute to severe shock and end-organ injury, respectively. Consistent with this, high plasma kynurenine levels associate with ICU delirium^51^. Preclinical models support inhibition of KMO as a strategy to reduce acute kidney injury in a mouse model of ischemia/reperfusion^52^ and multiorgan injury in rodent models of acute pancreatitis^50,53^. Together, these data support kynurenine metabolism among preclinical targets as a potential multifaceted therapeutic axis in CI, though further integrated studies will be necessary to prove utility.

This study has several limitations. The generalizability of these findings to other CI populations, including trauma and burn, or to less severely ill patients with infection outside of the ICU, remains unclear. Most analyses were performed at a single time point 2 days post-ICU admission. Limited blood volumes and a focus on rare cell subsets, like T_reg_ cells, prevented the use of certain assays like stable isotope tracing and extracellular flux analysis, which require larger cell numbers. We deferred expanding T_reg_ cells, as such protocols risk substantial metabolic confounding. We validated some of the transcriptomic findings experimentally (ROS and kynurenine), while others remain exploratory and hypothesis generating, particularly where effect sizes are modest and transcript abundances are low. In addition, effect sizes varied across experimental findings, reflecting both sepsis heterogeneity and its multifactorial nature, where no single pathway likely fully accounts for the observed metabolic remodeling.

Despite these constraints, our study used human biospecimens exclusively, included a diverse cohort of nonseptic ICU controls and generated a large scRNA-seq dataset from PBMCs in non-COVID CI. By focusing on ICU patients (with a median SOFA score of 9 in CI-Sep), these data complement previous large-scale human sepsis scRNA-seq studies that enrolled broader, less severely ill cohorts (for example, median SOFA 2–4 (ref. ^54^) and 5 (ref. ^55^)). While CI-Sep generally exhibited larger effect sizes than CI-NS, the directionality of changes was consistent between the two, suggesting overlapping mechanisms of metabolic dysfunction in CI.

Our results indicated that metabolic remodeling favored T_reg_ cell persistence and suppressive function (a shift broadly implicated in patient morbidity and mortality)^56^. These findings provide mechanistic insight into CD4^+^ T cell dysregulation in human CI and underscore the value of context-specific, cell subset-resolved analyses. More broadly, they highlight that newly acquired metabolic capacities, not just metabolic deficiencies such as impaired mitochondrial function^19,57,58^, may shape immune cell fate in CI and sepsis. These findings inform our understanding of immune dysfunction in human CI and suggest precision-based candidates for restoring immune homeostasis.

## Methods

### Human participants, clinical metrics and sepsis adjudication

All study protocols were approved by the Vanderbilt Institutional Review Board (IRB) (institutional protocol approval numbers 211462 and 191562). CI adults (≥18 years of age) were enrolled from the medical and surgical ICUs at Vanderbilt University Medical Center from 2022 to 2024 as part of The Sepsis ClinicAl Resource And Biorepository (SCARAB). Exclusion criteria included active exsanguination without hemostatic control or hemoglobin ≤7.5 g dl^−1^, ICU stay >48 h or cardiac arrest before enrollment, moribund state with imminent death, receipt of extracorporeal membrane oxygenation at the time of enrollment, pregnancy, medical trainees, in police custody, ICU admission solely for frequency of nursing care, or admission for uncomplicated diabetic ketoacidosis, drug overdose or alcohol withdrawal. For this analysis, we additionally excluded individuals receiving T cell-directed immunosuppression (such as tacrolimus, azathioprine and mycophenolate), immunomodulatory antineoplastic agents (such as pembrolizumab, ipilimumab and rituximab) and those with severe leukopenia with insufficient PBMCs for analysis, active hematologic malignancy or previous allogenic stem cell transplant.

Written informed consent was obtained from patients or surrogates. If informed consent could not be obtained owing to lack of patient capacity or inability to reach a listed surrogate, then patients were enrolled under an IRB-approved waiver of consent for minimal-risk research. Nonacutely ill healthy controls were recruited from bedside surrogates of enrolled ICU patients and provided written informed consent. Healthy controls were required to be ≥18 years of age; exclusion criteria included: pregnancy, presence of an acute infection, severe anemia, receipt of T cell-directed immunosuppression or receiving any immunomodulatory antineoplastic agents, presence of a hematologic malignancy, or previous allogeneic stem cell transplant. ICU participants were not compensated; healthy controls received USD40 for participation.

Peripheral blood was collected from patients in the ICU within 24 h of ICU admission (day 0) and then serially at days 2 and 7 after study enrollment. Unless otherwise stated, all experiments were performed with day-2 samples. Approximately 7–11 ml of blood were collected at each time point in citrate and EDTA vacutainer tubes, which were pooled for PBMC isolation. Clinical data, including demographics, comorbidities, vital signs, laboratory data, imaging reports, medications and flowsheet data were abstracted from the electronic medical record into a HIPAA-compliant REDCap database. We abstracted International Classification of Disease, Clinical Modification 10 (ICD-10-CM) diagnoses codes entered into the electronic medical record during the course of each patient’s hospital admission to identify comorbidities and calculate the weighted Elixhauser–van Walraven Comorbidity Index^59,60^. Healthy control participants contributed up to 50 ml of peripheral blood at a single time point; only age, sex and race/ethnicity were recorded in a HIPAA-compliant REDCap database.

Sepsis status was adjudicated post-hospitalization by a practicing physician intensivist (Extended Data Fig. 1). The presence of an infection was established based on positive bacterial cultures, infection-equivalent findings such as a visibly contaminated abdomen during laparotomy or through integrative clinical assessment in the absence of the former. End-organ dysfunction was assessed using Sepsis-3 criteria, defined as a SOFA score increase of ≥2 from the patient’s baseline^2^. Patients meeting both infection and organ-injury criteria as a result of that infection were classified as having sepsis (CI-Sep); all others were designated nonsepsis (CI-NS).

### Peripheral blood mononuclear cell isolation and storage

Fresh peripheral blood was diluted 1:2 with PBS and layered over 15 ml of Lymphoprep density gradient medium (STEMCELL Tech., cat. no. 18061) in SepMate-50 tubes (STEMCELL Tech., cat. no.85450). After density gradient centrifugation (1,200*g* for 15 min at 20 °C), the PBMC-containing upper layer was pelleted (400*g* for 10 min at 20 °C), RBC-lysed for 7 min with ACK buffer (Gibco, cat. no. A1049201), washed (300*g* for 10 min at 20 °C), filtered through a 70-μm strainer and washed a final time (300*g* for 10 min at 20 °C). Isolated PBMCs were resuspended in Bambanker Cell Freezing Medium (GC LYMPHOTEC, cat. no. BB02) at 2 million PBMCs per ml, aliquoted and slow frozen in a CoolCell FTS30 freezing container (Corning, cat. no. 432006) at −80 °C. PBMC aliquots were transferred to liquid nitrogen vapor phase storage within 72 h.

### Cryorecovery of PBMCs

PBMCs were thawed by rapid rewarming at 37 °C for 3 min and immediately diluted 1:5 in HPLM (Gibco, cat. no. A4899101) supplemented with 10% dialyzed fetal bovine serum (dFBS) (Sigma-Aldrich, cat. no. F0392) and 100 U ml^−1^ of penicillin plus 100 μg ml^−1^ of streptomycin (P/S) (Gibco, cat. no. 15140122). Cells were centrifuged at 300*g* for 5 min at 20 °C, washed twice in the same medium and filtered through a 35-μm cell strainer cap during the final wash to remove debris. PBMCs were resuspended in HPLM with 10% dFBS and P/S for immediate use in downstream experiments.

### Flow cytometry

**Extended Data Fig. 8:**
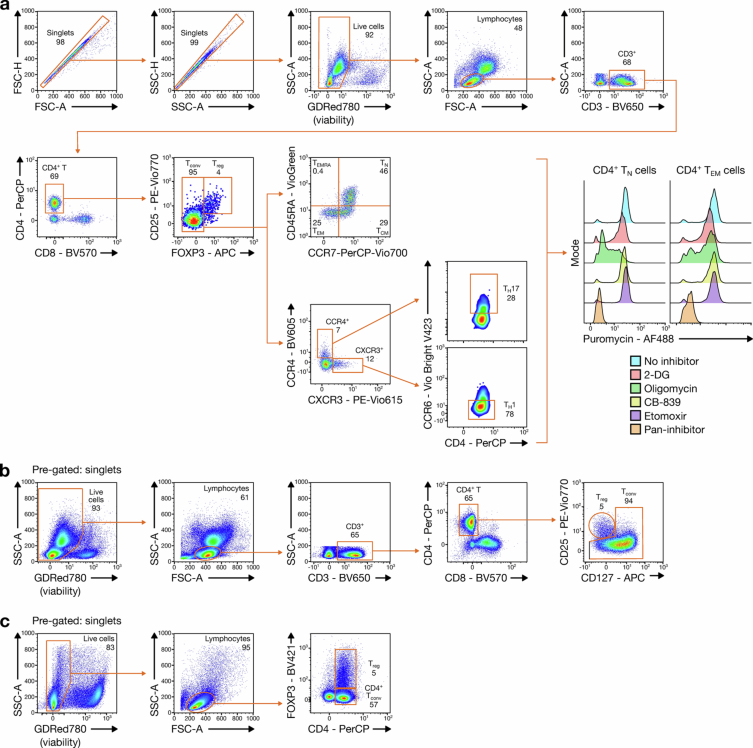
Representative flow cytometry gating strategies. **a**, Gating strategy for the identification of FSC^lo^SSC^lo^CD3^+^CD4^+^ T_N_ cells (FOXP3^−^CD45RA^+^CCR7^+^), T_EM_ cells (FOXP3^−^CD45RA^−^CCR7^−^), T_CM_ cells (FOXP3^−^CD45RA^−^CCR7^+^), T_H_1 cells (FOXP3^−^CXCR3^+^CCR4^−^CCR6^−^), T_H_17 cells (FOXP3^−^CXCR3^−^CCR4^+^CCR6^+^), and T_reg_ cells (FOXP3^+^CD25^+^) using surface and intracellular staining. Representative anti-puromycin staining with and without metabolic inhibitors for NHC CD4^+^ T_N_ and T_EM_ cells is shown. **b**, Gating strategy for the identification of unstimulated FSC^lo^SSC^lo^CD3^+^CD4^+^ T_conv_ cells (CD25^lo^CD127^hi^) and T_reg_ cells (CD25^hi^CD127^lo^) using surface staining only. **c**, Gating strategy for the identification of T_conv_ cells (FSC^lo^SSC^lo^CD4^+^FOXP3^−^) and T_reg_ cells (FSC^lo^SSC^lo^CD4^+^FOXP3^+^) in CD3 + CD28 + CD2 Ab stimulation experiments.

Cells were distributed in 96-well V-bottom microplates (Corning, cat. no. 3894), pelleted (300*g* for 5 min at 4 °C) and washed twice in cold PBS. All cell staining was conducted in 50-μl volumes. Cells were stained with Ghose Dye Red 780 Fixable Viability Dye (Cell Signaling, cat. no. 18452, diluted 1:2,000) and Human TruStain FcX solution for Fc receptor blocking (BioLegend, cat. no. 422302, diluted 1:40) in PBS for 30 min at 4 °C. Cells were washed with flow staining buffer (cold PBS with 2% FBS) and stained for surface markers (antibodies listed in Extended Data Table 2) in flow staining buffer for 30 min at 4 °C followed by two additional washes. In some experiments, intracellular markers were evaluated. Cells were fixed and permeabilized using 100 μl of Fix/Perm reagent (Tonbo Biosciences, cat. no. TNB-0607-KIT) for 30 min at room temperature followed by two washes in permeabilization buffer (Tonbo Biosciences, cat. no. TNB-1213-L150). Cells were stained for intracellular markers (antibodies in Extended Data Table 2) in permeabilization buffer for 30 min at 4 °C followed by two washes in permeabilization buffer. Data were acquired on a Miltenyi MACSQuant Analyzer 16 flow cytometer and exported as MQD files, which were analyzed in FlowJo v.10.9.0 (Becton Dickson & Company). Representative gating is shown (Extended Data Fig. 8).

### Puromycin-incorporation assay

We measured metabolic dependencies and capacities using a flow cytometric puromycin-incorporation assay adapted from the SCENITH method^61^. After cryothaw, PBMCs were rested for 3 h cells at 37 °C in HPLM with 10% dFBS and P/S to allow for metabolic recovery. For each sample, single metabolic inhibitors were added in separate wells for 30 min at 37 °C: glucose-dependent pathway inhibitor 2-deoxy-d-glucose (2-DG) (Cayman Chemical, cat. no. 14325, 100 mM), mitochondrial ATP synthase inhibitor oligomycin A (Cayman Chemical, cat. no. 11342, 1.5 μM), glutaminase 1 (GLS1) glutaminolysis inhibitor CB-839 (MedChemExpress, cat. no. HY-12248, 10 μM) or carnitine palmitoyltransferase-1 (CPT-1) fatty acid oxidation inhibitor Etomoxir (Sigma-Aldrich, cat. no. E1905, 5 μM). Control wells with vehicle or a full inhibitor cocktail (ATP extinction control) were included. Cells were pulsed with puromycin (Sigma-Aldrich, cat. no. P8833, 10 μM) for 40 min at 37 °C, washed in cold PBS and prepared for flow cytometry with intracellular detection of puromycin by antibody staining as described above. Metabolic dependencies and capacities were calculated from the geometric MFI of puromycin as originally reported^61^. Where indicated, cells were pretreated for 3 h or 24 h at 37 °C in HPLM with 10% dFBS and P/S with l-kynurenine (MedChemExpress, cat. no. HY-104026, 10 μM), l-leucine (MedChemExpress, cat. no. HY-N0486500, 500 μM), GSK180 (MedChemExpress, cat. no. HY-112179, 10 μM), phthalic acid (MedChemExpress, cat. no. HY-I0508, 100 μM), FX-11 (Millipore-Sigma, cat. no. 427218, 20 μM) or MitoTEMPO (MedChemExpress, cat. no. HY-112879, 10 μM) before the puromycin-incorporation assay.

### Mitochondrial mass, membrane potential and ROS

Mitochondrial mass, mitochondrial membrane potential and mitochondrial ROS were measured using MitoTracker Green FM (Invitrogen, cat. no. M46750), TMRE (Invitrogen, cat. no. T669) and MitoSOX Green superoxide indicator (Invitrogen, cat. no. M36006), respectively. Cryorecovered PBMCs were resuspended in HPLM with 10% dFBS and P/S, rested for 30 min at 37 °C and treated with either MitoTracker Green FM (25 nM), TMRE (40 nM), or MitoSOX Green (500 nM) for 30 min at 37 °C. Cells were washed twice with cold flow staining buffer and prepared for flow cytometry as described above.

### Ex vivo restimulation for CD4^+^ T cell function

Cryorecovered PBMCs were resuspended in HPLM with 10% dFBS and P/S and stimulated with ImmunoCult Human CD3/CD28/CD2 T Cell Activator (STEMCELL Tech., cat. no. 10970, 1:20 dilution) for 24 h at 37 °C. Brefeldin A (GolgiPlug, BD, cat. no. 555029, 1:1,000 dilution) was added for the final 5 h to enable intracellular cytokine detection. Cells were analyzed by flow cytometry as described above. Where indicated, cells were treated with low-dose oligomycin (Cayman Chemical, cat. no. 11342, 10 nM), GSK180 (MedChemExpress, cat. no. HY-112179, 10 μM) or MitoTEMPO (MedChemExpress, cat. no. HY-112879, 10 μM) for the full 24 h stimulation.

### Kynurenine uptake assay

Intracellular kynurenine uptake was measured as previously described^35^ with modifications. Cryorecovered PBMCs were incubated in HPLM with 10% dFBS and P/S for 1 h at 37 °C. Cells were washed twice with warm PBS and stained with Fc-block, viability dye and cell surface antibodies in PBS for 30 min at room temperature. Cells were then washed once with prewarmed 37 °C HBSS and resuspended in 37 °C HBSS containing l-kynurenine (MedChemExpress, cat. no. HY-104026, 100 μM) for 5 min. Uptake was quenched with 100 μl IC Fixation Buffer (Invitrogen, cat. no. 00-8333) for 30 min at room temperature, washed twice with flow staining buffer and analyzed by flow cytometry. l-kynurenine was detected by its intrinsic spectral properties using a 405-nm excitation (violet laser) and a 450/50-nm emission filter (V1 channel, MACSQuant Analyzer 16). Where indicated, LAT1 transport inhibitor BCH (MedChemExpress, cat. no. HY-108540, 10 mM) was added at time of cell plating or a saturating quantity of LAT1 substrate l-leucine (MedChemExpress, cat. no. HY-N0486, 5 mM) was added at the time of l-kynurenine treatment as controls for receptor specificity. Where indicated, HBSS with l-kynurenine chilled to 4 °C was added to cells on ice for the 5-min pulse to discriminate l-kynurenine uptake versus cell surface binding.

### LC–MS/MS quantification of kynurenine in cell culture medium

To determine the pre-existing availability of l-kynurenine in the media preparations used in experiments where modulations included adding exogenous l-kynurenine or a kynurenine-3-monooxygenase inhibitor (KMOi), we performed quantitative liquid chromatography with tandem mass spectrometry (LC–MS/MS). l-kynurenine levels were tested in fresh HPLM, fresh HPLM plus 10% dFBS and P/S, and HPLM plus 10% dFBS and P/S stored for 30 days at 4 °C. Then, 1 ml of −80 °C 80:20 methanol:water was added to the 330 µl of the medium sample in a 1.7-ml tube and vortexed vigorously. Then, 20 nmol of internal standard (^13^C-1-Lactate, Millipore-Sigma) was spiked into each sample. Cells were then placed in −80 °C freezer to extract for 15 min. After extraction, any insoluble debris was pelleted at 16,000*g* for 10 min at 4 °C. The supernatant containing extracted metabolites was then transferred to a new 1.7-ml tube. Extracts were then dried under N_2_. Dried samples were resuspended in 50 µl 3:2 mobile buffer A: mobile buffer B (see below) and transferred to 1.7-ml tubes. Samples were then centrifuged at 16,000*g* for 10 min at 4 °C to remove any insoluble debris. A standard curve was generated using 20 nmol of internal standard (^13^C-1-Lactate) in samples containing either 0, 3.75, 7.5, 15 or 30 nmol of a natural isotope kynurenine standard. Then, 18 µl of the sample was then chromatographed with a Shimadzu LC system equipped with a 100 × 2.1 mm, 3.5-μm particle diameter XBridge Amide column (Waters). Mobile phase A: 20 mM NH_4_OAc, 20 mM NH_4_OH and 5% acetonitrile in H_2_O, pH 9.45 (pH with NH_4_OH); mobile phase B: 100% acetonitrile. With a flow rate of 0.45 ml min^−1^ the following gradient was used: 2.0 min, 95% B; 3.0 min, 85% B; 5.0 min, 85% B; 6.0 min, 80% B; 8.0 min, 80% B; 9.0 min, 75% B; 10 min, 75% B; 11 min, 70% B; 12 min, 70% B; 13 min, 50% B; 15 min, 50% B; 16 min 0% B; 17.5 min, 0% B; and 18 min, 95% B. The column was equilibrated for 3 min at 95% B between each sample. Scheduled multiple reaction monitoring (MRM) was conducted in negative and positive mode with a detection window of 120 s using an AB SCIEX 6500 QTRAP with the following analyte parameters: *m/*z 209 → 94 (positive mode, retention time: 6.3 min) for l-kynurenine; *m/z* 90 → 43 (negative mode, retention time of 6.0 min) for ^13^C-1-lactate internal standard. All analytes were quantified via LC–MS/MS using the standard curve.

### Single-cell library preparation and RNA sequencing

Cryopreserved PBMCs were rapidly thawed at 37 °C for 3 min, diluted dropwise into 7 ml of room temperature HPLM with 10% dFBS and centrifuged at 300*g* for 5 min. Cells were washed once with HPLM with 10% dFBS and once in cold PBS with 0.04% bovine serum albumin (BSA), with the final wash passed through a 35-μm cell strainer (Falcon, cat. no. 352235). Cells were resuspended in cold PBS with 0.04% BSA at 1,000 cells per μl. Single-cell RNA-seq libraries were generated using the Chromium Next GEM Single Cell 5’ HT Kit v2 (10x Genomics) targeting 20,000 cells per sample. Libraries were sequenced on an Illumina NovaSeq 6000 (S4 flow cell, PE150) to a target depth of 50,000 reads per cell. Raw data were processed with Cell Ranger (v.7.1.0) using the GRCh38-2020-A genome assembly with the intron inclusion option enabled.

### ScRNA-seq preprocessing, quality control, integration and modeling

Raw feature barcode matrices from Cell Ranger were processed through a custom Nextflow workflow executed within Docker containers on AWS HealthOmics. Ambient RNA and technical artifacts were removed using CellBender remove-background (v.0.3.0)^62^. Learning rates (0.0000005–0.00005) and training epochs (150–300) were iteratively tuned; defaults were used for all other parameters.

CellBender outputs were filtered using Seurat (v.4.3.0)^63^ to exclude cells with >20% mitochondrial content or <400 or >5,000 detected features. Doublets were identified and removed using scDblFinder (v.1.13.10)^64^ using temporary SCTransform normalization and shared nearest neighbor clustering (2,000 highly variable genes (HVGs), 30 principal components and resolution 0.5), which were discarded after doublet removal. Sample-level metadata were added, and cleaned matrices were converted to AnnData using the sceasy package (v.0.0.7)^65^.

Filtered per-sample data were merged, normalized (target_sum = 1 × 10^4^) and log-transformed using Scanpy (v.1.9.5)^66^. The top 5,000 HVGs were selected using the ‘seurat_v3’ method. Data were integrated and modeled using scVI-tools (v.1.0.4)^67^. The scVI model was trained using n_hidden = 128, n_latent = 30, n_layers = 2, negative binomial likelihood (gene_likelihood = ‘nb’) with gene level dispersion, dropout rate of 0.1, AdamW optimizer and a learning rate of 0.001. Training proceeded for up to 400 epochs with early stopping. Modeling incorporated categorical covariates (Biologic_Sex), continuous covariates (percent.mt, nCount_RNA, and percent.ribosomal) and batch correction (batch_key).

### scRNA-seq clustering, visualization and annotation

Scanpy (v.1.9.5) was used to create a *k*-nearest neighbors (*k*NN) graph (scanpy.pp.neighbors) using the scVI latent space (use_rep = ‘X_scVI’, n_neighbors = 30). Clustering was performed with the Leiden algorithm (scanpy.tl.leiden, resolution = 1). DEGs for each cluster were identified using scVI’s differential_expression function with denoised expression values. Cell types were annotated using canonical marker genes with the Azimuth Human PBMC core reference map as the primary guide^68^, supplemented by literature-defined markers and expert consensus. Clusters lacking clear marker dominance were iteratively subclustered (resolutions between 0.2 and 2.0) until high-confidence annotations could be assigned. Clusters exhibiting two distinct, nonoverlapping gene expression profiles were excluded as potential doublets. A small B cell cluster unique to sample 10069-MS-0033 expressing canonical markers of chronic lymphocytic leukemia (*ROR1*, *FMOD* and *CD5*) and identical heavy- and light-chain V-segments (IGHV3-66 and IGKV1D-39) was excluded as a probable subclinical neoplasm. Final annotations were provided at two levels: celltype_coarse for major lineages and celltype_fine for granular subpopulations.

### Cell proportionality

Cell lineage proportions were quantified using Scanpro (v.0.2)^69^ applied to NHC, CI-NS and CI-Sep samples with both celltype_coarse and celltype_fine cell lineage annotations.

### Differential gene expression and pathway analysis

Pseudobulk profiles were generated from single-cell data using the Python implementation of the decoupleR package (v.1.6.0)^70^ by summing counts per cell type across within each sample, retaining profiles from ≥20 cells and with ≥1,000 total counts. Differential expression was performed using PyDESeq2 (v.1.40.2)^71^ with low-expression genes removed (filter_by_expr function, min_count=3, min_total_count = 25). Visualization of per-sample gene expression was performed using pseudobulk counts normalized to CPM and log-transformed. Visualization of single-cell gene expression was performed using Scanpy-normalized and log-transformed single-cell counts. Normalized, scaled pseudobulk gene expression profiles were used to select the top 2,000 HVGs (Seurat v3 method) followed by hierarchical clustering using Ward’s linkage based on Euclidean distances.

Over-representation analysis of significantly upregulated genes (FDR-adjusted *P* < 0.05) was performed with decoupleR using the GO:BP collection. GSEA^72^ was performed on pseudobulk differential gene expression results (Wald statistic) using GSEApy (v.1.1.4)^73^ with 1,000 permutations and the KEGG 2021 Human and Hallmark 2020 libraries, using a fixed random seed. NES and FDR-adjusted *P* values were used to score pathways. A binary matrix indicating leading-edge gene membership across significantly enriched gene sets (FDR-adjusted *P* < 0.05) was constructed and pairwise Jaccard similarity between pathways was calculated based on shared leading-edge genes. Pathway activity for a human pan-tissue AHR gene signature^45^ was quantified using the AUCell method^74^ implemented in decoupleR.

### Flux balance analysis

FBA was conducted using the Compass package (v.0.9.10.2)^75^. For each condition and cell subset, single-cell transcriptomes were normalized to CPM (scanpy.pp.normalize_total) and aggregated into tab-delimited input files. Compass was run with the IBM CPLEX optimization engine using the Recon2 consensus metabolic reconstruction^76^ and default parameters (penalty-diffusion knn, lambda 0, num-neighbors 30) with microclusters of 25 cells. Reaction penalty scores were negative log-transformed to yield values representing reaction flux potential and filtered to remove low-confidence or invariant reaction. For bidirectional reactions, all reactions were modeled but only positive fluxes were retained for visualization. Reaction activity was standardized as *z*-scores or calculated as Cohen’s *d* effect sizes for visualization and comparison between conditions.

### Statistics and reproducibility

Statistical analyses were performed in Python (v.3.9) using scipy.stats (v.1.11.4), scikit-posthocs (v.0.9.0) and statsmodels (v.0.14.1). Two-sided Mann–Whitney *U*-tests were used for two-group comparisons and Kruskal–Wallis tests with Dunn’s post hoc testing for multigroup comparisons. Spearman correlations were used for associations with LOWESS smoothing (fraction = 0.8) with 1,000 bootstrap resamples applied where indicated. Patient summary statistics (median, IQR and *P* values) were generated using tableone (v.0.9.1).

For scRNA-seq analyses, logit-transformed proportion testing used an empirical Bayes ANOVA (scanpro); differential expression used the Wald test with Benjamini–Hochberg correction (DESeq2); over-representation analysis used a one-tailed Fisher’s exact test with Benjamini–Hochberg correction; gene-set enrichment used a permutation-based Kolmogorov–Smirnov statistic with Benjamini–Hochberg correction (pyGSEA); and Compass flux comparisons used two-sided Mann–Whitney *U*-tests with Benjamini–Hochberg correction. Effect sizes were represented as Cohen’s *d* values derived from the pooled s.d. Bar graphs show means with error bars representing s.e.m. Boxplots follow standard conventions (median line, IQR box boundaries and whiskers extending to the minimum and maximum values within 1.5 × IQR).

No statistical method was used to predetermine sample size but our sample sizes are similar to those reported in previous publications^54,55^. For flow cytometric assays reporting mean intensity values, including the puromycin-incorporation assay, cell populations with <25 detected cells were excluded because a mean could not reliably be estimated. One scRNA-seq sample (10069-MS-0036) was excluded before analysis after grossly failing quality control metrics. Some clinical variables were unavailable for specific patients in the electronic medical record and were therefore not included. The investigators were not blind to the sample group assignments while completing the analysis. Most data were generated from ≥2 independent experimental replicates and shown as pooled data, with limited analyses performed in a single replicate (Figs. 2a,c,d, 6f,g and 7k,l and Extended Data Figs. 3b and 7b–f).

### Reporting summary

Further information on research design is available in the Nature Portfolio Reporting Summary linked to this article.

## Online content

Any methods, additional references, Nature Portfolio reporting summaries, source data, extended data, supplementary information, acknowledgements, peer review information; details of author contributions and competing interests; and statements of data and code availability are available at 10.1038/s41590-025-02390-6.

## Supplementary information

Reporting Summary

Supplementary Table 1 Pseudobulk DESeq2 results.

Supplementary Table 2 Over-representation analysis results.

Supplementary Table 3 GSEA results.

Supplementary Table 4 Compass FBA results.

## Source data

Source Data Fig. 1 Source data for figures.

Source Data Fig. 2 Source data for figures.

Source Data Fig. 3 Source data for figures.

Source Data Fig. 4 Source data for figures.

Source Data Fig. 6 Source data for figures.

Source Data Fig. 7 Source data for figures.

Source Data Extended Data Fig. 2 Source data for figures.

Source Data Extended Data Fig. 3 Source data for figures.

Source Data Extended Data Fig. 4 Source data for figures.

Source Data Extended Data Fig. 7 Source data for figures.

## Figures and Tables

**Extended Data Table 1:**
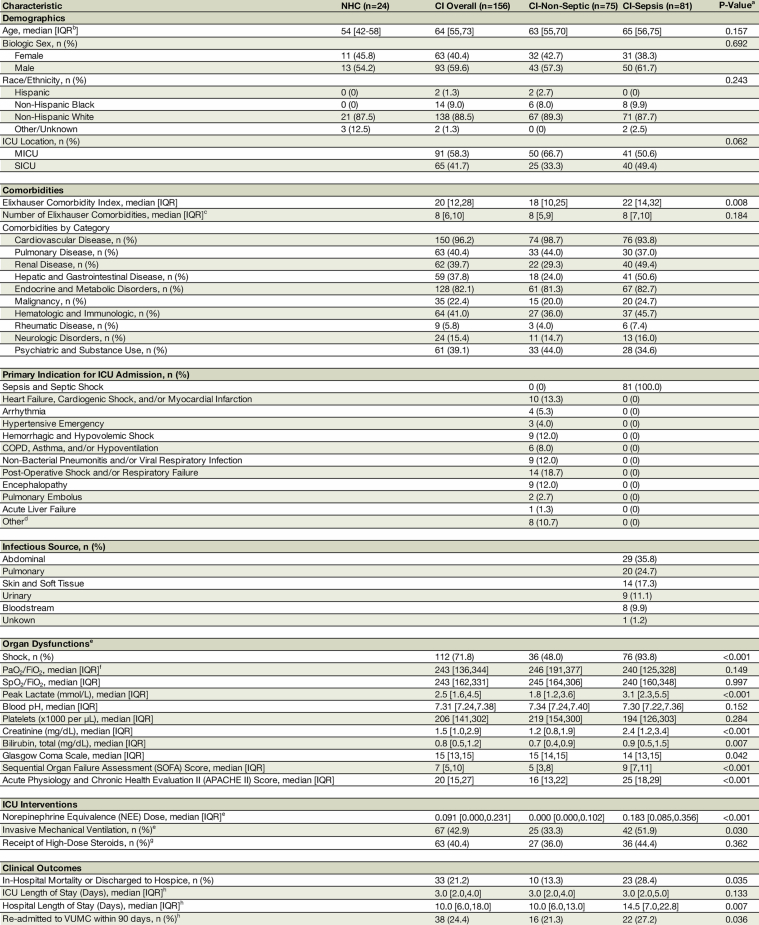
Baseline characteristics and incident ICU clinical data for study participants

^a^P-values are calculated using the Chi-squared test (for categorical variables) or the Mann–Whitney U-test (for continuous variables) comparing CI-Non-Septic and CI-Sepsis.

^b^Interquartile Range [25th, 75th percentile].

^c^Out of a total of 31 sampled comorbidities.

^d^Includes pneumothorax/pneumomediastinum, myxedema coma, SVC syndrome, malignant compressive airway compromise, and abdominal compartment syndrome.

^e^Values reflect the first 24 h of ICU admission.

^f^PaO2/FiO2 ratio was calculated only for patients with available arterial blood gas (ABG) measurements.

^g^Defined as ≥ 40 mg prednisone (or equivalent) administered within the first 72 h after ICU admission.

^h^Values reflect only patients who survived to hospital discharge.

**Extended Data Table 2:**
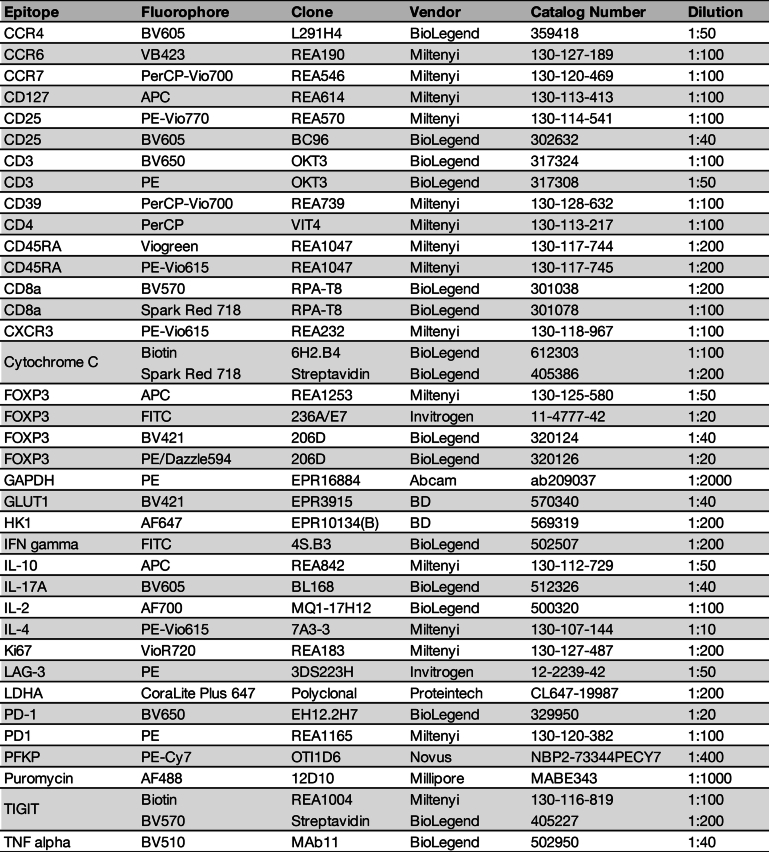
Flow cytometry antibodies

Detailed information for the flow cytometry antibodies used throughout this study.

## Data Availability

Single-cell RNA-seq data supporting this study have been deposited in the Gene Expression Omnibus (GEO) under accession no. GSE290679 with FASTQ files available in the Database of Genotypes and Phenotypes under accession nos. phs004258.v1.p1 (ICU patients) and phs004377.v1.p1 (healthy donors). Source data are provided with the paper, except certain clinical data that could be potentially uniquely identifying. Further information and requests for data should be directed to, and will be fulfilled by, the corresponding authors J.C.R. and M.T.S. Source data are provided with this paper.

## Code availability

Scripts associated with the data analysis in this manuscript have been deposited at https://github.com/StierLab/2025-CD4T-sepsis.
