# Supplementary material for: Metabolic adaptations rewire CD4+ T cells in a subset-specific manner in human critical illness with and without sepsis
Source: Nat Immunol. 2026 Jan 15;27(2):236–49. doi: 10.1038/s41590-025-02390-6 (PMC12864044; doi:10.1038/s41590-025-02390-6)
Supplement: Supplementary file 1 — Reporting Summary [file 41590_2025_2390_MOESM1_ESM.pdf]

nature portfolio

Corresponding author(s): Jeffrey C. Rathmell, PhD and Matthew T. Stier, MD, PhD

Last updated by author(s): 12/1/2025

Reporting Summary

Nature Portfolio wishes to improve the reproducibility of the work that we publish. This form provides structure for consistency and transparency in reporting. For further information on Nature Portfolio policies, see our Editorial Policies and the Editorial Policy Checklist.

Statistics

For all statistical analyses, confirm that the following items are present in the figure legend, table legend, main text, or Methods section.

n/a	Confirmed	
☐	☒	The exact sample size (n) for each experimental group/condition, given as a discrete number and unit of measurement
☐	☒	A statement on whether measurements were taken from distinct samples or whether the same sample was measured repeatedly
☐	☒	The statistical test(s) used AND whether they are one- or two-sided<br>*Only common tests should be described solely by name; describe more complex techniques in the Methods section.*
☒	☐	A description of all covariates tested
☐	☒	A description of any assumptions or corrections, such as tests of normality and adjustment for multiple comparisons
☐	☒	A full description of the statistical parameters including central tendency (e.g. means) or other basic estimates (e.g. regression coefficient) AND variation (e.g. standard deviation) or associated estimates of uncertainty (e.g. confidence intervals)
☐	☒	For null hypothesis testing, the test statistic (e.g. F, t, r) with confidence intervals, effect sizes, degrees of freedom and P value noted<br>*Give P values as exact values whenever suitable.*
☒	☐	For Bayesian analysis, information on the choice of priors and Markov chain Monte Carlo settings
☒	☐	For hierarchical and complex designs, identification of the appropriate level for tests and full reporting of outcomes
☐	☒	Estimates of effect sizes (e.g. Cohen's d, Pearson's r), indicating how they were calculated

Our web collection on statistics for biologists contains articles on many of the points above.

Software and code

Policy information about availability of computer code

Data collection: n/a

Data analysis:
Python==3.9
jupyterlab==4.0.10
Scanpy==1.9.5
scVI-tools==1.0.4
CellBender==0.3.0
scDblFinder==1.13.10
sceasy==0.0.7
Compass==0.9.10.2
CPLEX==22.1.1.0
PyDESeq2==1.40.2
decoupleR==1.6.0
AUCell (included in decoupleR package)
GSEApy==1.1.4
Scanpro==0.2
matplotlib==3.6.3
seaborn==0.11.2
scikit-posthocs==0.9.0
statsmodels==0.14.1
scipy.stats==1.11.4

```
tableone==0.9.1
R==4.3.0
Seurat==4.3.0
reticulate==1.28
PyTorch==CUDA 11.7 optimized
JAX==CUDA 11 support
Nextflow DSL2
AWS HealthOmics, S3
FlowJo==10.9.0

Code: https://github.com/StierLab/2025-CD4T-sepsis
```

For manuscripts utilizing custom algorithms or software that are central to the research but not yet described in published literature, software must be made available to editors and reviewers. We strongly encourage code deposition in a community repository (e.g. GitHub). See the Nature Portfolio guidelines for submitting code & software for further information.

Data

Policy information about availability of data

All manuscripts must include a data availability statement. This statement should provide the following information, where applicable:

- Accession codes, unique identifiers, or web links for publicly available datasets
- A description of any restrictions on data availability
- For clinical datasets or third party data, please ensure that the statement adheres to our policy

Single-cell RNA-seq data supporting this study have been deposited in the Gene Expression Omnibus (GEO) under accession number GSE290679 with FASTQ files available in the Database of Genotypes and Phenotypes (dbGaP) under accession numbers phs004258.v1.p1 (ICU patients) and phs004377.v1.p1 (healthy donors). Source data are provided with the paper, except certain clinical data that could be potentially uniquely identifying. Further information and requests for data should be directed to, and will be fulfilled by, the corresponding authors J.C.R. and M.T.S.

Research involving human participants, their data, or biological material

Policy information about studies with human participants or human data. See also policy information about sex, gender (identity/presentation), and sexual orientation and race, ethnicity and racism.

Reporting on sex and gender	Participants were not excluded based on sex or gender. Aggregate data on sex (self-reported and/or obtained from the electronic health record) is reported in Extended Data Table 1. Data stratification by sex was performed where indicated in the text. In all other analyses, data from all participants was pooled.
Reporting on race, ethnicity, or other socially relevant groupings	Aggregate data on race and ethnicity (self-reported and/or obtained from the electronic health record) is reported in Extended Data table 1. No analyses were performed using race, ethnicity, or other socially relevant groupings.
Population characteristics	Full details are described in Extended Data Table 1.
Recruitment	Participants were recruited from the medical and surgical intensive care units (ICUs) at Vanderbilt University Medical Center (VUMC) as part of the Sepsis Clinical Resource and Biorepository (SCARAB) study between 2022 and 2024. Critically ill patients were included if they were ≥18 years old and met criteria for ICU admission, with adjudication into critically ill non-septic (CI-NS) or critically ill septic (CI-Sep) groups based on Sepsis-3 criteria and the algorithm in Extended Data Figure 1. Non-acutely ill healthy controls (NHC) were recruited from bedside surrogates of enrolled ICU patients to provide a community control cohort. Full inclusion and exclusion criteria are listed in the Methods.
Ethics oversight	The protocols for SCARAB and healthy participants were reviewed, approved, and monitored by the Institutional Review Board at Vanderbilt University Medical Center under the study IDs #211462 and #191562, respectively.

Note that full information on the approval of the study protocol must also be provided in the manuscript.

Field-specific reporting

Please select the one below that is the best fit for your research. If you are not sure, read the appropriate sections before making your selection.

☒ Life sciences ☐ Behavioural & social sciences ☐ Ecological, evolutionary & environmental sciences

For a reference copy of the document with all sections, see nature.com/documents/nr-reporting-summary-flat.pdf

Life sciences study design

All studies must disclose on these points even when the disclosure is negative.

Sample size	No formal power calculation was performed due to the exploratory nature of this study. Sample sizes were targeted based on prior human CD4 T cell studies in critical illness and sepsis, as well as practical limitations of patient sample availability.

Data exclusions	For flow cytometry, certain samples contained too few cells in one or more CD4+ T cell subsets for reliable analysis (e.g., <25 cells). In these cases, the affected subsets were excluded, but the rest of the sample was retained. In scRNA-seq, one NHC sample (10069-MS-0036) was excluded due to grossly failing quality control. During annotation, clusters with distinct, non-overlapping gene expression profiles from two different cell lineages were removed as potential doublets. Additionally, in CI-Sep sample 10069-MS-0033, a small B cell population expressing chronic lymphocytic leukemia markers (ROR1, FMOD, CD5) and sharing identical heavy/light chains was excluded as a likely subclinical neoplasm. Some clinical data were absent due to not being collected as part of the usual clinical care for an individual patient (e.g. arterial blood gas to measure PaO2/FiO2 ratio).
Replication	Each patient sample served as a biologic replicate. Most data were generated from ≥2 independent experimental replicates and shown as pooled data, with limited analyses performed in a single replicate (Fig. 2a,c,d; Fig. 6f,g; Fig. 7k,l; Extended Data Fig. 3b; Extended Data Fig. 7b–f). The scRNA-seq analysis was conducted on samples from multiple patients, and key findings were validated using orthogonal approaches, including flow cytometry and functional assays.
Randomization	Patient samples were selected based on predefined clinical criteria for sepsis and non-septic controls. Given the observational nature of this study, randomization was not applicable.
Blinding	Data processing and computational analyses were conducted using standardized pipelines to minimize bias. Where possible, analyses were completed without knowledge of the group assignments of each sample, but no formal blinding protocol was implemented.

Reporting for specific materials, systems and methods

We require information from authors about some types of materials, experimental systems and methods used in many studies. Here, indicate whether each material, system or method listed is relevant to your study. If you are not sure if a list item applies to your research, read the appropriate section before selecting a response.

Materials & experimental systems

n/a	Involved in the study
☐	☒ Antibodies
☒	☐ Eukaryotic cell lines
☒	☐ Palaeontology and archaeology
☒	☐ Animals and other organisms
☒	☐ Clinical data
☒	☐ Dual use research of concern
☒	☐ Plants

Methods

n/a	Involved in the study
☒	☐ ChIP-seq
☐	☒ Flow cytometry
☒	☐ MRI-based neuroimaging

Antibodies

Antibodies used	Full antibody details including the protein target, clone, fluorophore conjugate, vendor, catalog number, and dilution are listed in Extended Data Table 2.
Validation	All antibodies used in this study were commercially available at the time of study performance from established vendors with experience in flow cytometric antibody production. Antibody validation was performed by the listed vendor as indicated in the manufacturer documentation and available on the manufacturer websites.

Plants

Seed stocks	N/A
Novel plant genotypes	N/A
Authentication	N/A

Flow Cytometry

Plots

Confirm that:

☒ The axis labels state the marker and fluorochrome used (e.g. CD4-FITC).

☒ The axis scales are clearly visible. Include numbers along axes only for bottom left plot of group (a 'group' is an analysis of identical markers).

☒ All plots are contour plots with outliers or pseudocolor plots.

☒ A numerical value for number of cells or percentage (with statistics) is provided.

Methodology

Sample preparation	PBMC were isolated from fresh whole blood from critically ill or healthy participants via density gradient centrifugation and cryostored. Thawed PBMC were either plated for experimental testing or stained immediately for flow cytometry depending upon the intended analysis. Samples were stained with a fixable live/dead viability dye and Fc blocking reagents followed by cell surface antibodies and, where applicable, were fixed/permeabilized and stained with intracellular antibodies. Samples were then immediately analyzed on a flow cytometer. Full details including incubation times and conditions are provided in the Methods section.
Instrument	Miltenyi MACSQuant Analyzer 16 Flow Cytometer
Software	FlowJo version 10.9.0
Cell population abundance	N/A
Gating strategy	Samples were initially gated to exclude doublets by FSC-H/FSC-A and SSC-H/SSC-A followed by gating for viable cells (Ghost Dye Red 780 negative cells). The lymphocyte fraction was gated as FSC-A low and SSC-A low. Gating for each individual population was performed as outlined in Extended Data Table 3.

☒ Tick this box to confirm that a figure exemplifying the gating strategy is provided in the Supplementary Information.
